# The relationship between the Ewald sphere and exit wave explored using focal series electron micrographs

**DOI:** 10.1107/S2052252525010796

**Published:** 2026-01-01

**Authors:** J. Bernard Heymann

**Affiliations:** a National Cryo-EM Program, Cancer Research Technology Program Frederick National Laboratory for Cancer Research, Leidos Biomedical Research Inc. Frederick MD21701 USA; Boston University School of Medicine, USA

**Keywords:** electron microscopy, cryoEM, image formation, contrast transfer function, aberration function, reconstruction

## Abstract

CryoEM reconstructions must be performed along the Ewald spheres to achieve resolutions beyond the projection approximation limit. The linear theory of image formation models the scattering from the specimen and focusing by the objective lens as two conjugate Ewald spheres that correspond to the two halves of the contrast transfer function (CTF). A focal series of micrographs mimics the focal gradient inherent in the Ewald wavefront, and its 3D Fourier transform provides a visual representation of the conjugate Ewald spheres. To better understand the nature of the Ewald spheres, focal series of graphene oxide as a thin specimen and platinum–iridium as a strongly scattering specimen were acquired. An algorithm was developed to fit and correct the CTF of the whole focal series. Correcting for the one half of the CTF flattens one of the Ewald spheres in the 3D transform, while doubling the curvature of the other. This yields the best representation of the Ewald sphere and thus the ‘exit wave’, and can be performed on every single micrograph. The 2D reconstruction as the sum of the partial CTF corrected transforms recovers missing information in the individual micrographs and increases the signal-to-noise ratio. Iterative refinement of the reconstructed image against the amplitudes of the original focal series recovers the second Ewald sphere only at frequencies where the two spheres overlap, but introduces artifacts. The conclusion is that the correct way to account for the Ewald spheres in cryoEM is to apply the two halves of the CTF to a particle image and incorporate the two results into their corresponding spheres within the cryoEM reconstruction.

## Abstract 2

The Ewald sphere is inherent in image formation in the electron microscope and must therefore be part of the reconstruction of cryoEM maps. This study examines linear image-formation theory to show how Ewald sphere information derives from focal series micrographs and relates to the exit wave. It concludes by suggesting the proper way to correct for the contrast transfer function for cryoEM reconstruction.

## 1. Introduction

While cryoEM has achieved remarkable results (Yip *et al.*, 2020 ▸; Sottatipreedawong *et al.*, 2025 ▸), we are still refining our understanding of image formation in the electron microscope to extract every bit of information from the micrographs. The spherical scattering inherent in the interaction between the electron beam and the specimen is at the heart of any theory of image formation. The spherical geometry leads to the ‘exit wave’ emerging at the bottom of the specimen, and the coherent wave passing through the back focal plane known as the Ewald sphere. The extent of the Ewald sphere in the electron beam direction is a function of the focal gradient through the specimen and thus its thickness (DeRosier, 2000 ▸; Heymann, 2023 ▸). At high acceleration voltages, the Ewald sphere can be approximated by a 2D plane, leading to the ‘projection approximation’ commonly used in cryoEM. However, with thick specimens and beyond a resolution of 2–3 Å, the Ewald sphere must be considered to calculate cryoEM reconstructions (Wolf *et al.*, 2006 ▸; Heymann, 2023 ▸).

**Figure 1:**
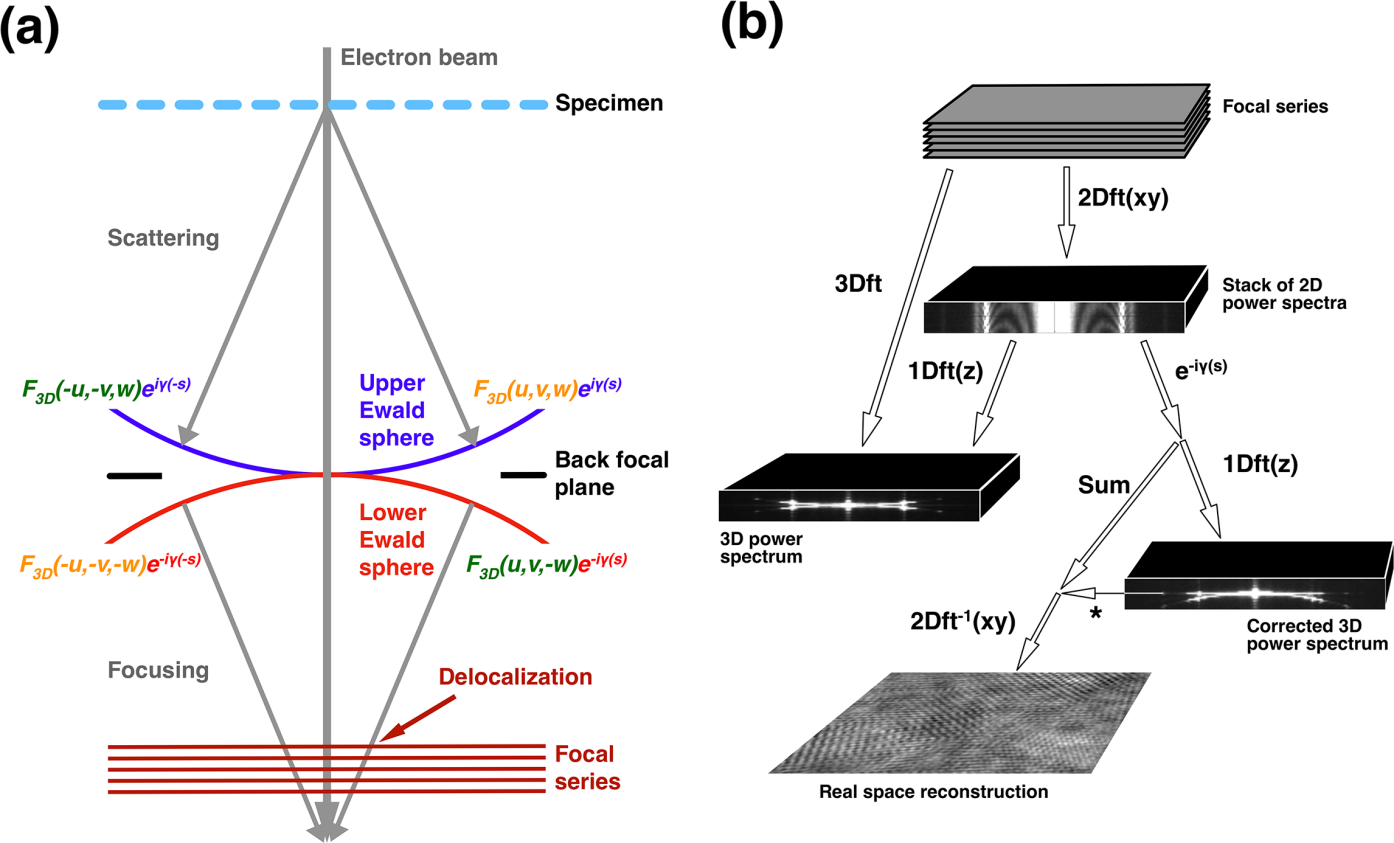
(*a*) The scattering–focusing geometry in the electron microscope leads to two spherical peaks in intensity: the conjugate Ewald spheres. The lower Ewald sphere (red) is the conjugate (negative sign in the exponent) of the upper Ewald sphere (blue). However, the structure factor in the upper left is the conjugate of that in the lower right: 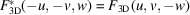 (green) and the structure factor in the upper right is the conjugate of that in the lower left: 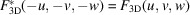 (orange), adhering to Friedel symmetry. A focal series intersects the focusing rays at different heights, leading to a delocalization of where the electrons hit on the detector. The latter results in the contrast transfer function (CTF) that must be corrected for reconstruction. (*b*) A stack of focal series micrographs Fourier transformed in 2D gives a stack of power spectra showing the change in CTF with a change in focus. A 3D Fourier transform reveals two spheres with their peak ridges coinciding with the Ewald spheres. Performing a correction for one half of the CTF {exp[−*i*γ(*s*)]} on the stack of 2D transforms, and transforming the result only in the *z* (or *w*) direction, flattens one Ewald sphere and doubles the curvature of the other. The middle section of this 3D transform (indicated by *) is equivalent to the sum of the corrected 2D transforms, and on back transformation in 2D gives the focal series reconstruction with appropriate real-space phases.

The Ewald sphere describes the spherical wavefront where the scattering from the atoms at different heights interferes constructively [Fig. 1 ▸(*a*)]. Away from the wavefront, the interference is destructive so that the signal is suppressed. In diffraction from crystals, this leads to the suppression of reflections that do not lie on or close to the Ewald sphere. The greater the range of heights of the atoms, *i.e.* the thickness of the specimen, the narrower the width of constructive interference. The heights of the atoms relate to the focus and only the atoms in the same plane are at the same focus.

Focusing the scattered beam forms a mirror geometry of the scattering, so that the interference effect is reflected in a second, conjugate Ewald sphere [Fig. 1 ▸(*a*)]. The concept of a second Ewald sphere is not new, as it was presented in the context of light scattering and focusing by Hopkins (1953 ▸) and adopted for electron microscopy in Wolf *et al.* (2006 ▸). Because the typical mode of transmission electron microscopy can be seen as in-line Fresnel holography, Wade also presented it as two conjugate spheres (Wade, 1992 ▸). In cryoEM reconstruction accounting for the Ewald spheres, the integration is along both spheres in frequency space (Wolf *et al.*, 2006 ▸; Russo & Henderson, 2018 ▸; Zivanov *et al.*, 2018 ▸; Heymann, 2023 ▸).

The focal gradient resulting in the Ewald spheres can be mimicked by a focal series of micrographs, where the 3D Fourier transform exhibits two conjugate spheres [Fig. 1 ▸(*b*)] (Taniguchi *et al.*, 1991 ▸; Kimoto *et al.*, 2012 ▸, 2013 ▸; Ishizuka & Kimoto, 2016 ▸; Heymann *et al.*, 2023 ▸). This is the result of the inherent Friedel symmetry of the 3D transform, consistent with the geometry of scattering and focusing in the electron microscope (Heymann, 2023 ▸). Just as the upper Ewald sphere derives from the coherency of the scattered electrons, the lower Ewald sphere reflects the coherency of the focused electrons. This is consistent with the two halves of the contrast transfer function (CTF). It is essential to properly correct for the CTF by accounting for the two Ewald spheres in cryoEM reconstructions to recover the best possible resolution from a given set of micrographs (Heymann *et al.*, 2023 ▸; Heymann, 2023 ▸).

The ‘exit wave’ referenced in many papers should be the pattern of electrons emerging at the bottom of the specimen (van Dyck & Coene, 1987 ▸), subject to both elastic and inelastic multiple scattering. This has given rise to many ‘phase-retrieval’ algorithms (Schiske, 1968 ▸, 2002 ▸; Gerchberg & Saxton, 1972 ▸; Fienup, 1982 ▸; Frank, 1972 ▸; Kirkland *et al.*, 1980 ▸; van Dyck *et al.*, 1996 ▸; Op de Beeck *et al.*, 1996 ▸), where the mere generation of a complex image is commonly taken to be the exit wave. One would assume that the Ewald sphere wavefront should be equivalent to the Fourier transform of the exit wave, in the same way that a diffraction pattern or power spectrum is calculated by Fourier transformation of a micrograph.

In the context of cryoEM reconstructions, the correct integration of the micrograph data into the 3D volume is of great importance in achieving high resolution. The most prevalent accounting for the Ewald sphere in reconstruction is by the simple insertion method (Wolf *et al.*, 2006 ▸; Heymann, 2023 ▸) commonly adopted in many software packages. An alternative interpretation is based on associating single side bands with the left and right parts of the Ewald sphere (Russo & Henderson, 2018 ▸). As these approaches are significantly different, it is pertinent to resolve the matter and establish the correct way.

Here, I use focal series of micrographs to examine the relationships between the Ewald sphere, the 3D Fourier transform of a focal series, exit waves, CTF correction and the proper way to perform cryoEM reconstructions (following on from Heymann *et al.*, 2023 ▸). In the theory part, I show that partial CTF corrections could be performed on every individual micrograph, and that the summation of the corrected micrographs in the focal series gives a 2D reconstruction with an increased signal-to-noise ratio (SNR). This is then the basis for a correct approach to Ewald-sphere-based reconstruction in cryoEM. The different calculations to explore this are shown in Fig. 1 ▸(*b*).

## 2. Theory

### 2.1. Preliminaries

The diffraction pattern of any particle scattering (either in electron microscopy or X-ray crystallography) in the Fraunhofer or projection approximation is closely modeled by the Fourier transform of the projection through the specimen, according to the projection-slice theorem (Bracewell, 1956 ▸). In the projection approximation, the curvature of the scattering wavefront is ignored, and it is taken to be a 2D plane, equivalent to the central section of a 3D Fourier transform. Therefore, to consider the spherical scattering geometry that results in the curvature of the Ewald sphere we must examine it in the 3D context. Focusing aims at mirroring the scattering angles to form an image, thus establishing a second, conjugate Ewald sphere [Fig. 1 ▸(*a*)]. There is a correspondence between the geometry of scattering and focusing, and the frequency-space representation of the 3D distribution of atoms in the specimen, so that the coordinate frames can be superimposed (Heymann, 2023 ▸). The coordinate frame is chosen such that the electron beam propagates in the *z* direction, perpendicular to the *xy* plane. Given the real-space atom coordinates as vectors {*x*, *y*, *z*}, the change in focus through the specimen corresponds to the *z* coordinate. Fourier transformation gives the frequency-space representation with the coordinates {*u*, *v*, *w*}, where the *w* coordinate relates to the transform over all the atomic *z* coordinates, as well as the gradient of focus as part of the CTF. In the following theory I present the mathematical expression of these concepts.

### 2.2. The Ewald sphere projection for a single micrograph

The projection approximation commonly used in cryoEM relates the image intensity and the central section of the object potential in frequency space modified by the CTF as (Unwin & Henderson, 1975 ▸)
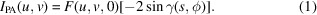
where γ(*s*, ϕ) is the phase shift imposed by the CTF at the spatial frequency *s* = (*u*^2^ + *v*^2^)^1/2^ and an angle ϕ = tan^−1^*v*/*u*. If we then consider the Ewald sphere, we want to have an equation that approximates this at small scattering angles. The appropriate formula models the Ewald sphere as the superposition of the spherical scattering and focusing geometries on the object representation in frequency space (Heymann, 2023 ▸). This superimposes on the 3D object transform with the third dimension in the scattering context given by the change in focus through the specimen. The intensity is therefore proportional to the 3D frequency-space potential, *F*(*u*, *v*, *w*), modified by the aberration functions corresponding to the conjugate spherical geometry of scattering and focusing (Hoppe, 1970 ▸; Han *et al.*, 1995 ▸):
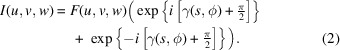
If we set *w* = 0 and assume symmetric terms only, we obtain equation (1) (Russo & Henderson, 2018 ▸). This is consistent with a common derivation described as the linear approximation for image formation (Hoppe, 1970 ▸; Kirkland *et al.*, 1980 ▸; Saxton, 1995 ▸; Meyer *et al.*, 2002 ▸). This also relates the two halves of the CTF to the two Ewald spheres [Fig. 1 ▸(*a*)].

We model the phase shift for every atom, *a*, using the major terms for the CTF (Unwin & Henderson, 1975 ▸; Typke & Radermacher, 1982 ▸),
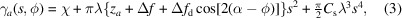
where λ is the electron wavelength, χ is the constant phase shift associated with amplitude contrast (Typke & Radermacher, 1982 ▸), *z*_*a*_ is the coordinate of the atom in the electron-beam direction relative to the center of the specimen, Δ*f* is the average defocus at the center of the specimen, Δ*f*_d_ is the focal deviation for astigmatism at an angle α, compared with the angle of the structure factor, ϕ = tan^−1^*v*/*u*, and *C*_s_ is the spherical aberration coefficient. The aberration function can be expanded to include other terms (Zernike, 1934 ▸; Typke & Köstler, 1976 ▸), but these do not significantly influence the results in this study (Supplementary Table S1). Note that we can separate the focus difference associated with an atom from the average aberration function:
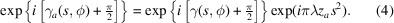
The last term is the Fresnel propagator that associates the spherical scattering geometry with the atomic *z* coordinate (Hoppe, 1970 ▸). We define the 3D object potential in frequency space based on the original derivation by Born (1926 ▸) widely used in both X-ray crystallography (Ten Eyck, 1977 ▸; Agarwal, 1978 ▸) and electron microscopy (Hoppe, 1970 ▸; Sorzano *et al.*, 2014 ▸; Heymann, 2023 ▸):
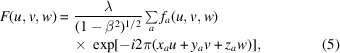
where *f*_*a*_(*u*, *v*, *w*) is the amplitude of the cross section of atom *a* at coordinates {*x*_*a*_, *y*_*a*_, *z*_*a*_} and the prefactor is the relativistic electron wavelength, with β the electron speed relative to light speed (Rez, 2003 ▸).

The two terms in equation (2) constitute two superimposed volumes associated with the two Ewald spheres [Fig. 1 ▸(*a*)]. Thus, we can write the two volumes for the upper and lower Ewald spheres that differ only in the signs of the average aberration function and the Fresnel propagator:
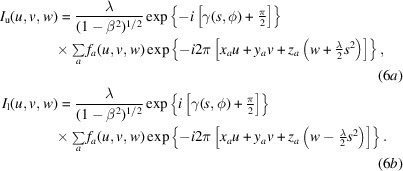
As shown in equation (4), we can separate the terms in the exponential kernel, so that we isolate the integral over the *z* coordinate. If we assume there is an effective thickness *t* with a uniform distribution of *z* coordinates, we can integrate over this thickness to obtain a normalized sinc function
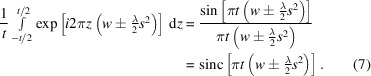
This means that the signal is spread out along the *w* axis through convolution with a sinc function inversely related to the sample thickness. The sinc function oscillates infinitely, but beyond the first node the intensity is small. Therefore, the ‘width’ used here refers to the main peak of the normalized sinc function or zeroth-order spherical Bessel function of the first kind, *J*_0_(π*x*) = (sinπ*x*)/π*x*, in the range *x* = [−1, 1]. For many specimens the distribution of *z* coordinates is not uniform, affecting the effective thickness and thus the width of the sinc function. Therefore, the appropriate value for *t* depends on the shape of the molecule (Heymann, 2023 ▸). For example, a spherically shaped molecule has an effective diameter that is the height of a cylinder with the same radius and volume, which is significantly less than the diameter of the molecule. Also, the thicker the specimen, the narrower the width of the sinc function, so that most of the coherent information is in the peak. This sinc function imposes a filter on the frequency-space representation such that the power spectrum is
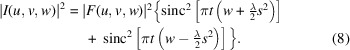
This produces two spherical curves in the 3D power spectrum [Fig. 1 ▸(*b*)], where the peaks or ridges are taken as the Ewald spheres. The phases resulting from the summations in equation (6), however, depend on the distribution of the atoms.

If we multiply equation (6*a*) with one half of the CTF and take the peak of the Ewald sphere at *w* = −(λ/2)*s*^2^, we recover the information at one Ewald sphere:
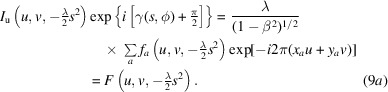
The result is a 2D image because we eliminated the effect of the *z* coordinates, ‘flattening’ the Ewald sphere. If we also multiply equation (6*b*) with the same aberration function, we double its phase as well as the phase associated with atomic *z* coordinates:
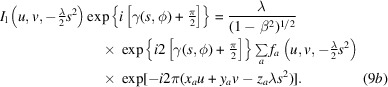
This doubles the curvature of the sphere, so that it separates from the other sphere at a lower frequency [Fig. 1 ▸(*b*); this is illustrated in Section 4]. We therefore recover the information of one Ewald sphere at higher frequencies, and the sum of the two Ewald spheres at lower frequencies. Thus, we can obtain the Ewald sphere information at high resolution from a single micrograph but corrupted with considerable noise. This partial recovery can then be enhanced by averaging over multiple micrographs to eliminate most of the noncoherent information (treated as noise) that includes the terms in equation (9*b*). Equation (9) is well known in phase retrieval (see Bhat & Datta, 2022 ▸) and underlies all image-reconstruction techniques in some form. In cryoEM, the CTF correction according to equation (1) mainly accounts for the part where two Ewald spheres overlap, while the coherence at higher frequencies ensures proper reconstruction if the integration is along the Ewald spheres (Heymann *et al.*, 2023 ▸).

### 2.3. The 3D Fourier transform of a focal series of micrographs

The 3D transform of a focal series of micrographs gives us a 3D context in which we can examine the Ewald spheres [Fig. 1 ▸(*b*)]. The appearance of the spherical curves in the 3D Fourier transform is a result of the integration in the direction of the electron beam. If we choose a reference image (usually the image in the middle of the focal series), the structure factors in the other images are phase-shifted by the Fresnel propagator relative to this image,
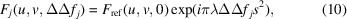
where ΔΔ*f*_*j*_ is the focal offset relative to the reference image. If we Fourier transform the stack of 2D transforms in the third dimension, *i.e.* in the direction of focus, the 3D structure factor is
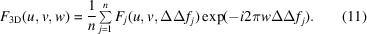
We now substitute equation (10) into equation (11),
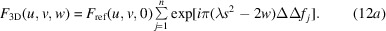
The 3D transform has inherent Friedel symmetry, so that we can write the equivalent equations [Fig. 1 ▸(*a*)]
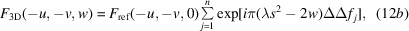

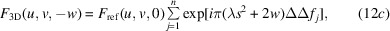

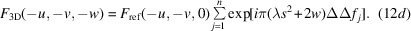
The full equation for the 3D transform is given by the superposition of the offsets in the positive and negative directions:
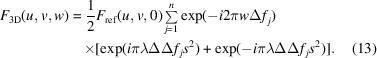
As in equation (7), we can approximate the summation by an integral over the range of focus, Δ*f*_r_ = ΔΔ*f*_max_ − ΔΔ*f*_min_:
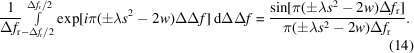
These are the spherical curves or ridges in the 3D Fourier transform [Fig. 1 ▸(*b*)]. Equation (14) is similar to the integral in equation (7), with maxima at *w* = ±(λ/2)*s*^2^, but the distribution of focus is now known, resulting in a defined width of the sinc function with the first node at *w* = ±1/Δ*f*_r_. Therefore, in the 3D Fourier transform, the widths of the spherical curves are only about two pixels, while for the Ewald sphere it is a function of the specimen thickness. The side lobes of the sinc function are very weak and thus do not contribute significantly to the 3D transform. This indicates that the idea that the so-called nonlinear information (*i.e.* off the sinc-function ridge) can be uniquely retrieved by a focal series is highly unlikely. The only useful information is at the ridges of the sinc functions that correspond to the Ewald spheres.

### 2.4. Reconstruction from a focal series of micrographs

The aim of a reconstruction from a focal series is to obtain a complex image (*i.e.* with phases) that should better represent the specimen (by implication at the Ewald spheres). If we apply equation (9) to each micrograph, we retrieve the high-resolution information along one Ewald sphere, resulting in some phase retrieval. Accumulating this across the focal series enhances the signal relative to noise:
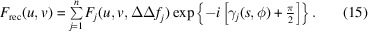
This reconstruction method is in essence the parabola method (Op de Beeck *et al.*, 1996 ▸) but without further iterative refinement.

Many methods in the literature attempt to refine the phases by iterative methods, comparing the intensities of the reconstruction with those of the original images (Coene *et al.*, 1992 ▸, 1996 ▸; Op de Beeck *et al.*, 1996 ▸; Saxton, 1994 ▸; Borisenko *et al.*, 2012 ▸; Ishizuka, 2013 ▸; Ophus & Ewalds, 2012 ▸). This is then taken to be the ‘exit wave’ and is supposed to represent the electron beam after exiting the specimen. The conceptual problems with this will be discussed later. However, such an iterative method would reveal how closely the reconstruction in equation (15) matches the actual image intensities. I therefore chose the iterative wavefunction reconstruction (IWFR; Allen *et al.*, 2004 ▸) to refine the real-space phases. The method uses the parabolic reconstruction in equation (15) in every iteration, followed by back Fourier transformation to obtain a complex real-space reconstruction with phases. The real-space amplitudes are then replaced with the original image intensities, and Fourier transformed as input for the next iteration of parabolic reconstruction. Convergence is assessed as the difference between the original image amplitudes and the real-space amplitudes of each parabolic reconstruction. Because we start from an initial parabolic reconstruction yielding real-space phases, the convergence is typically rapid, followed by very slow improvement over many iterations.

## 3. Materials and methods

All computational operations were performed with the *Bsoft* package (Heymann, 2018 ▸). A new program, *bfocal*, was developed to deal specifically with focal series.

### 3.1. Acquiring a focal series

Images of carbon film (Quantifoil), graphene oxide (GO) and platinum–iridium (PtIr) on carbon film were acquired at 200 kV with a CryoARM200 (Jeol) and a K3 direct detector (Gatan) at the minimum focal step size allowed on the microscope (nominally 37 Å) with the program *SerialEM* (Mastronarde, 2005 ▸). A 150 µm aperture was used in some imaging series and the energy-filter slit was 10 eV. Each series contains 20 images taken near true focus or somewhat underfocus (0.5–1 µm) at a nominal magnification of 150 000× with a pixel size of 0.16 Å. Each micrograph was taken over 6 s as a movie of 113 frames with a total dose of ∼4 e per pixel (∼156 e Å^−2^).

### 3.2. Micrograph processing

The movie frames were aligned and summed with the program *bseries*. The summed micrographs were further aligned with each other with the same program. To calculate 2D power spectra of the individual micrographs, 198 tiles of size 512 × 512 were extracted, Fourier transformed, converted to power spectra and averaged with the program *bctf*. To calculate 3D power spectra, 198 tiles of 512 × 512 × 20 were extracted, Fourier transformed, converted to power spectra and averaged with the program *bfft*. Orthogonal views were extracted with the program *b*3*v*. The full 3D Fourier transform from stacked images was also calculated with the program *bfft* to yield a complex image.

A mask to isolate the GO diffraction spots in the 3D Fourier transform of the stacked micrographs was constructed with the programs *beditimg*, *bimg* and *bsym*. Comparisons between images by Fourier ring correlation (FRC) were calculated with the program *bresolve*. Conversions from complex images to real, imaginary, intensity, amplitude and phase images were calculated with the program *bcomplex*.

### 3.3. Preparing the focal series power spectra for CTF fitting

The power spectra of the individual micrographs are noisy, making an estimation of the CTF difficult. We therefore devised a method to fit the CTF to the power spectra of the whole series at once, implemented in the program *bfocal*. To simplify the fitting, we rescaled the power spectra to eliminate most of the baseline (background) and envelope.

The complex elements of the Fourier transform are assumed to be composed of a coherent signal and random noise, with the signal modulated by the CTF:
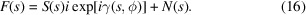
The power spectrum is the absolute square with a distinction between the even and odd terms of the aberration function:
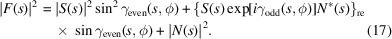
If we assume that the signal and noise are not correlated, the middle term is negligible, and we approximate the power as
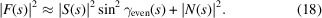
Thus, only the even terms impact the power spectrum. We then calculate a function that should correspond to the CTF:
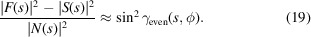
The signal and noise profiles are typically complicated and poorly represented by parametric functions. In general, these profiles are much smoother than the rapidly varying CTF and can therefore be estimated after smoothing. I devised the following method to approximate equation (19) (inspired by Barthel, 2007 ▸). The power spectrum is first smoothed with a Gaussian kernel (typically with σ ≈ 10 pixels), which gives an estimate of the noise power plus about half of the signal power: image 1. This is subtracted from the original power spectrum to give oscillations around zero: image 2. The absolute values of image 2 are then smoothed with the same Gaussian kernel and multiplied by π to obtain an approximation of the signal power: image 3. To obtain the final image consistent with equation (19), image 2 is divided by image 3 and truncated to [−1, 1]: image 4. The result is an image with the CTF maxima tending to 1 and minima tending to −1. The final noise power image is image 1 with half of the signal power image (image 3) subtracted. In the following sections we refer to image 4 as a stack of modified power spectra, *I*_mps_(*s*, ϕ).

### 3.4. Fitting the CTF

The objective function for fitting the CTF is a correlation coefficient calculated for the intensities in the modified power spectra, *I*_mps_(*s*, ϕ), up to a specified frequency *s*: 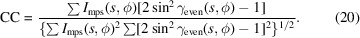
This generates a function where the oscillations traverse zero and thus emphasize the extrema. The absolute value of this function does not have meaning as it is dependent on several factors, including the amount of noise. The objective function is typically resolution-limited to avoid noise and low-frequency features. In our fits I used the resolution range 2.5–40 Å. The 2.5 Å cutoff eliminates the reflections of the GO and PtIr lattices to simplify the analysis.

The CTF is typically a rapidly varying function, rendering fitting prone to the local minimum problem. To avoid this, I devised an algorithm with multiple fitting steps implemented in the new program *bfocal*. Because we have a stack of 2D power spectra, we can extract a parallel (*xz* or *yz*) section and determine the initial defocus, defocus step size and spherical aberration (not affected by astigmatism). With defocus the most influential, I determined it first in the parallel section with a simple linear search. This was then followed by an iterative refinement of the three parameters on each section. Next, I included the astigmatism in a five-parameter iterative fit to the whole stack of modified 2D power spectra. To visually compare the result with the modified power spectra, half was replaced with the calculated CTF from the fit. For iterative fitting, I used the downhill simplex algorithm (Nelder & Mead, 1965 ▸) as adapted in *Bsoft* (Heymann, 2018 ▸) that requires good initial values for the parameters as obtained in our stepwise approach. More terms can be included in the CTF, but attempts to refine them indicated that their contributions are minor and the obtained values are likely to have large errors (Supplementary Section S1 and Table S1).

### 3.5. Focal series reconstruction

The focal series reconstruction was calculated according to equation (15), effectively the parabolic reconstruction method (Op de Beeck *et al.*, 1996 ▸) without iterative refinement, resulting in a complex frequency-space representation without Friedel symmetry (program *bfocal*). It was back Fourier transformed to yield the complex real-space image (program *bfft*).

To implement the Ewald sphere method of Russo & Henderson (2018 ▸), I examined the code from *RELION* 4.0.2 (https://github.com/3dem/relion) and wrote the equivalent as an option in the program *bfocal*. The key difference from equation (15) is that half of the micrograph is corrected with part of the CTF and the other half with the conjugate function. This creates a discontinuity between the two halves. They attempted to alleviate it by correcting the two halves in rotating sections. However, examination of the code revealed that the result is a single discontinuity at a hard-coded angle.

To quantify the SNR of the reconstruction as compared with the individual images, the spectral SNR (SSNR; Heymann, 2019 ▸, 2022 ▸) can be calculated both for the dose-fractionated series of each individual image as well as for the focal series reconstruction (program *bseries*). The SSNR is given by
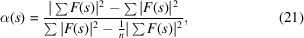
where *F*(*s*) is either an element of the Fourier transform of a single frame of the aligned movie of a dose-fractionated micrograph, or the micrograph after alignment and summation.

### 3.6. Iterative refinement of a focal series reconstruction

The iterative refinement of the reconstruction is based on the method of Allen *et al.* (2004 ▸) implemented in the program *bfocal*.

(i) A set of aberration functions for the stack of images is prepared using the CTF fit.
(ii) The stack of images is 2D Fourier transformed.
(iii) A parabolic reconstruction is performed using the set of aberration functions.
(iv) The reconstruction is multiplied with the conjugate of the set of aberration functions.
(v) These are back Fourier transformed to yield a set of complex images.
(vi) The amplitudes of these images are replaced by the original images.
(vii) The process is iterated through step (ii) until the change in root-mean-square difference between the original and reconstructed amplitudes decreases below a tolerance (typically set to 10^−6^) or a maximum number of iterations is reached.

## 4. Results

### 4.1. The 2D and 3D power spectra of a focal series

**Figure 2:**
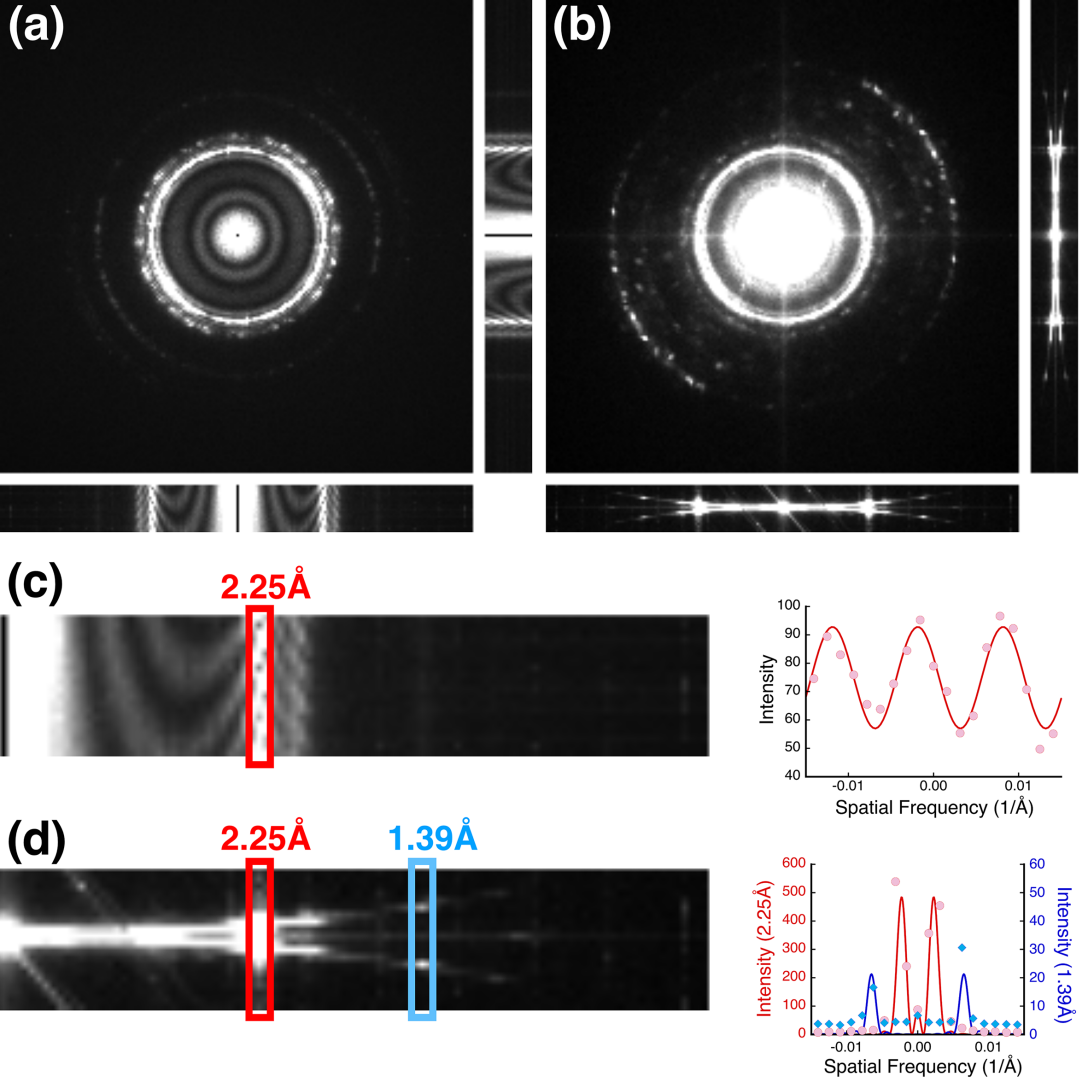
Focal series of platinum–iridium (PtIr) nanocrystals on carbon. (*a*) Orthogonal views of a stack of 2D power spectra of micrographs, showing bright peaks associated with the PtIr reflections and the oscillations of the contrast transfer function in the background from scattering by the carbon film. (*b*) Orthogonal views of the 3D power spectrum of the same stack of micrographs, with the coherent spheres resulting from the focus gradient evident in the lateral views. (*c*) Enlarged view of the *xz* transverse section through the 2D power spectra showing oscillations in the intensity of the 2.25 Å reflection (red rectangle) across the focal series with a period of 627 Å (close to the focal range of 608 Å). (*d*) Enlarged view of the *xz* section through the 3D power spectrum with reflections indicated at 2.25 Å (red rectangle) and 1.39 Å (blue rectangle), each modeled as dual sinc functions.

I first wanted to reproduce the 3D power spectrum of a focal series as was performed in previous studies (Taniguchi *et al.*, 1991 ▸; Kimoto *et al.*, 2012 ▸). On the CryoARM200 microscope, we are limited to a smallest focus step size of nominally 37 Å. However, in fitting the CTF it was discovered that the focal step is closer to 32 Å, indicating that it is important to include it as a fitting parameter (see Section 4.3). To observe the fading of the Ewald spheres in the 3D power spectrum, the sampling (pixel size) was chosen as 0.16 Å to give a frequency of 3.1 Å^−1^ at Nyquist. To test this, we collected 20 images in each focal series of three specimens: a carbon film of nominal thickness 400 Å, graphene oxide (GO) that is close to a single atomic layer, and platinum–iridium on carbon film (PtIr) as a more strongly scattering specimen. Each 2D image in the focal series was 2D Fourier transformed to obtain its power spectrum and then combined as a 3D volume to visualize the transverse sections [Fig. 1 ▸(*a*)]. Fig. 2 ▸(*a*) shows orthogonal views of this stack for a PtIr specimen (for the other specimens, see Supplementary Section S3). The PtIr nanocrystals are randomly oriented, resulting in mostly rings of spots in the power spectra rather than single lattice reflections. In the background the scattering from the carbon film shows the characteristic oscillations associated with the CTF. These oscillations persist through the PtIr reflections [Fig. 2 ▸(*c*)], with the period associated with the range in focus (∼640 Å). Treating the original stack of micrographs as a 3D volume and performing a 3D Fourier transform shows two spherical curves [Fig. 2 ▸(*b*)]. The peaks or ridges on these curves can be modeled as sinc functions with their first nodes at one-pixel offsets [Fig. 2 ▸(*d*)]. The offset from the plane then results in the oscillations across the 2D power spectra [Fig. 2 ▸(*c*)].

### 4.2. Shifting the graphene oxide reflection in the 3D transform to the center generates a simple projection

**Figure 3:**
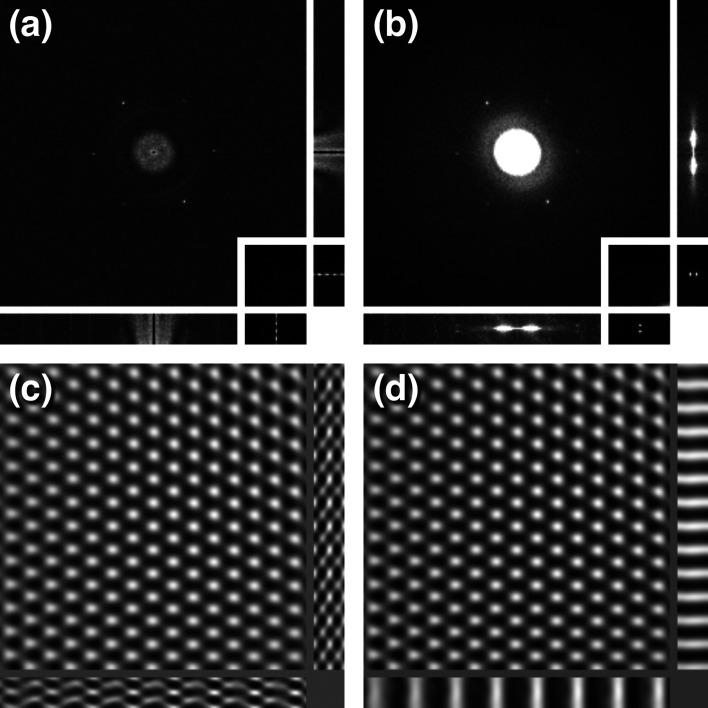
Focal series of single-layer graphene oxide. (*a*) Orthogonal views of the 2D power spectra near true focus. The inset shows the top left diffraction spot at 2.13 Å, with a periodic variation that reflects the offset of the sphere. (*b*) Orthogonal views of the 3D power spectrum. In the 3D power spectrum, the two spots fall on the two spheres, offset to a frequency of 0.0027 Å^−1^ (∼2 pixels). The inset shows the well separated reflections of the top left spot. (*c*) The six reflection spots on the upper sphere were masked and back transformed, showing oscillations in *z* consistent with the periodicity in the inset in (*a*). (*d*) The whole transform was shifted to the central section (−2 pixels), giving a constant density in the *z* direction as expected from a simple projection.

Graphene oxide was chosen as a very thin specimen (effectively the width of a single atom) with a simple diffraction pattern. The stack of 2D power spectra from a focal series of GO shows the six 2.13 Å reflections characteristic of the hexagonal lattice [Fig. 3 ▸(*a*)]. The variation in intensity of the six reflections results from the oscillations in the CTF. These oscillations can be seen in the inset in Fig. 3 ▸(*a*), and are consistent with the focal range. The 3D power spectrum of the stack exhibits well separated reflections on the two spheres [Fig. 3 ▸(*b*)]. This illustrates the narrowing of these spheres, as the Ewald spheres for a single-atom layer are expected to be much wider.

To obtain phase information, the full stack of 20 micrographs was 3D Fourier transformed. Two masks were generated to cover the diffraction spots at 2.13 Å from the GO lattice (size 20 × 20 × 3 voxels) at the two spheres (±2 voxels from the central section). The masked 3D transforms were back transformed to examine the appearance of the lattice across the focal series [Fig. 3 ▸(*c*)]. The oscillations in the *z* direction have a frequency consistent with the displacement of the reflection from the central section [consistent with the periodicity in the inset in Fig. 3 ▸(*a*)]. The 3D masked transforms were therefore shifted to the central section and back transformed [Fig. 3 ▸(*d*)]. It does not matter whether the spot on the upper or lower sphere is shifted; the same result is obtained. The lattice is now constant across the stack of images, representing a simple projection image. By shifting the reflection, the focal change through the series is compensated. This indicates that ‘flattening’ the sphere recovers projection information from the focal series.

### 4.3. Fitting the CTF in the focal series

**Figure 4:**
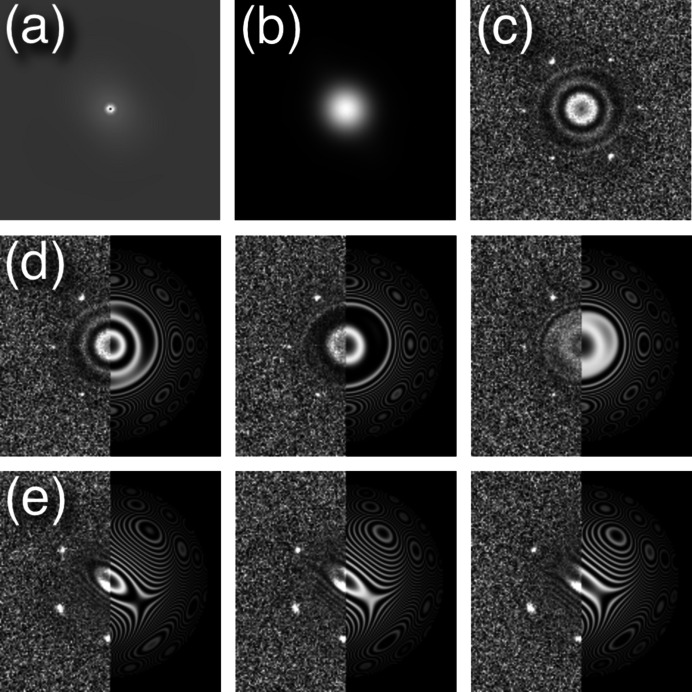
Fitting the even terms of the CTF to focal series of graphene oxide. (*a*)–(*c*) Preparation of a micrograph of graphene oxide for fitting the CTF. (*a*) Background. (*b*) Envelope. (*c*) Background subtracted and divided by the envelope. The CTF fit was limited to 2.5 Å to exclude the 2.13 Å reflections from the graphene lattice. (*d*) Fitting for an untilted beam. (*e*) Fitting for a beam tilted about 7.4 mrad. Three of the 20 modified power spectra in each series are shown in (*d*) and (*e*) in the left half with the fitted CTF in the right half.

The focal series power spectra present much more information than a single image and should be useful in determining the CTF of the whole series. While use of a focal series is not new (see Meyer *et al.*, 2002 ▸, 2004 ▸), the approach here makes better use of the series as a whole. Subtracting the background and adjusting for the scale of the envelope as slowly varying functions aid in simplifying the analysis [Figs. 4 ▸(*a*)–4 ▸(*c*)].

The rapid oscillations in the CTF complicate its analysis. I therefore developed a stepwise approach starting from *xz* and *yz* transverse sections through the modified focal power spectra (as described in Section 3). These sections show profiles mainly affected by three parameters: the average defocus for the middle image, the focus step size between images and spherical aberration. I only fitted two orthogonal transverse sections, but this could be expanded to more sections to better determine astigmatism from the variation in defocus. The fit was then refined including astigmatism for the whole focal series set of power spectra. Even with a thin specimen such as graphene oxide it was possible to fit the CTF [Figs. 4 ▸(*d*) and 4 ▸(*e*)], yielding reasonably consistent results.

One focal series was also recorded at a tilt to examine the effect on fitting [Fig. 4 ▸(*e*)]. The magnitude of tilt is mainly reflected in the astigmatism, which dominates over the residual astigmatism of the electron beam itself. The tilt angle is estimated from the additional astigmatism introduced (Barthel, 2007 ▸; Glaeser *et al.*, 2011 ▸). If we assume that the microscope is properly stigmated, the tilt angle is α_tilt_ ≈ Δ*f*_d,tilt_/(*C*_s_)^1/2^. For the case in Fig. 4 ▸(*e*), the tilt angle is estimated at 7.4 mrad.

It is possible to extend the fitting to more terms of the CTF (Supplementary Section S1). However, this adds more degrees of freedom that may not be justified. The results (Supplementary Table S1) show that the additional terms are minor, with large fitting errors. Consequently, these were not further explored.

One issue that did emerge from the more comprehensive fitting is the interdependence of the three main parameters: defocus, spherical aberration and amplitude contrast (Supplementary Section S2). It is possible to find different combinations of these terms that fit the data well enough to support correction of the micrographs. In these, the uncertainties are often at the lower frequencies, while fits are better at the higher frequencies. What this shows is that the fitted focus values should not be considered to be accurate reflections of the true defocus, but only working parameters for CTF correction.

### 4.4. Reconstructing platinum–iridium on carbon from a focal series

**Figure 5:**
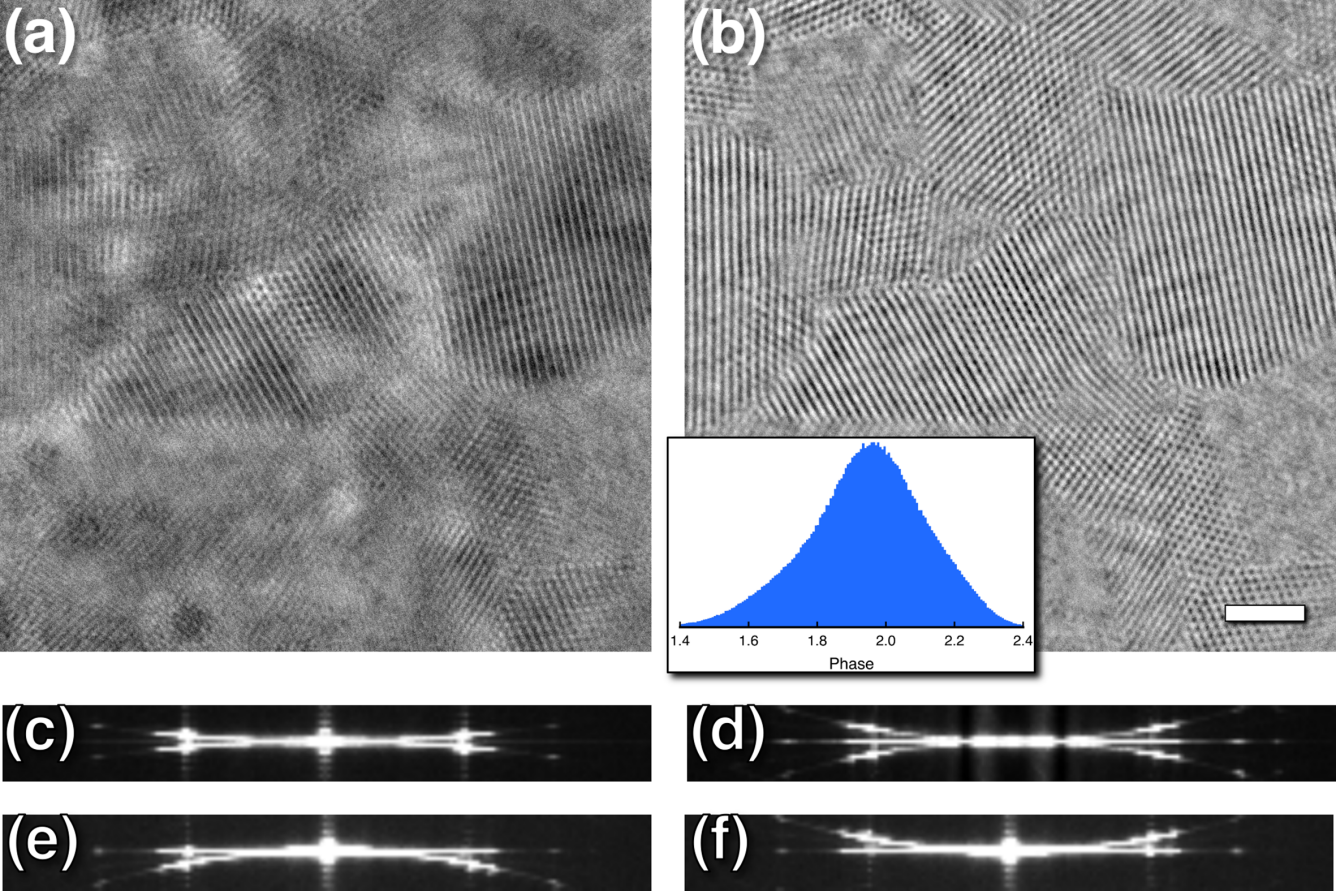
Part of a parabolic reconstruction from a focal series of micrographs of PtIr. (*a*) Amplitude image of a tile extracted from the larger reconstruction. (*b*) Phase image corresponding to (*a*) with a distribution peaked at ∼1.95 radians (112°). The inset shows the phase distribution. Scale bar: 20 Å. (*c*) 3D power spectrum of the focal series. (*d*) 3D power spectrum after applying the full CTF [equation (1)], producing a combination of the results in (*e*) and (*f*). (*e*) 3D power spectrum of the focal series after applying the first half of the CTF [equation (2)] as for the parabolic reconstruction, flattening one curve and doubling the curvature of the other. The flat plane corresponds to the real-space 2D images in (*a*) and (*b*). (*f*) 3D power spectrum of the focal series after applying the conjugate (second half) of the CTF [equation (2)].

PtIr was chosen as a strong scatterer to attempt a better understanding of focal series reconstruction. Focal series were acquired with and without the energy-filter slit, with little difference in the power spectra indicating a negligible contribution from inelastic scattering (Supplementary Section S4). The CTF fitting to the focal series 2D power spectra [Fig. 2 ▸(*a*)] was limited to frequencies below 2.5 Å to avoid the intense reflections for the heavy metals and use the signal from the carbon film. The CTF was fitted as for the carbon and graphene grids, but with an amplitude contrast of 0.38 appropriate for platinum (Typke & Radermacher, 1982 ▸). The parabolic reconstruction was performed using the fitted CTF parameters, yielding a complex image. Figs. 5 ▸(*a*) and 5 ▸(*b*) present the amplitude and phase images, respectively, of a tile extracted from the reconstruction of the full-size focal series. The amplitude image shows variation in density likely following the specimen thickness, while the phase variation reveals more detailed structural information (see Supplementary Section S5 for the real and imaginary images).

The original spherical curves in the 3D Fourier transform of the focal series [Fig. 5 ▸(*c*)] convert to one flattened curve and two exaggerated curves on the correction of each image with equation (1) [Fig. 5 ▸(*d*)]. If only one half of the CTF is applied [as in equation (9)], it converts to one flattened plane and one exaggerated sphere in the 3D Fourier transform [Fig. 5 ▸(*e*)]. Applying the conjugate aberration function flattens the other sphere [Fig. 5 ▸(*f*)]. The parabolic reconstruction is then the central 2D plane in Fig. 4 ▸(*e*), eliminating the parts of the other sphere that curve away from the plane at high frequencies. The conjugate reconstruction retrieves the conjugate Ewald sphere in the same way. Thus, the spheres carry different Ewald sphere information where they separate that needs to be accounted for in reconstruction.

**Figure 6:**
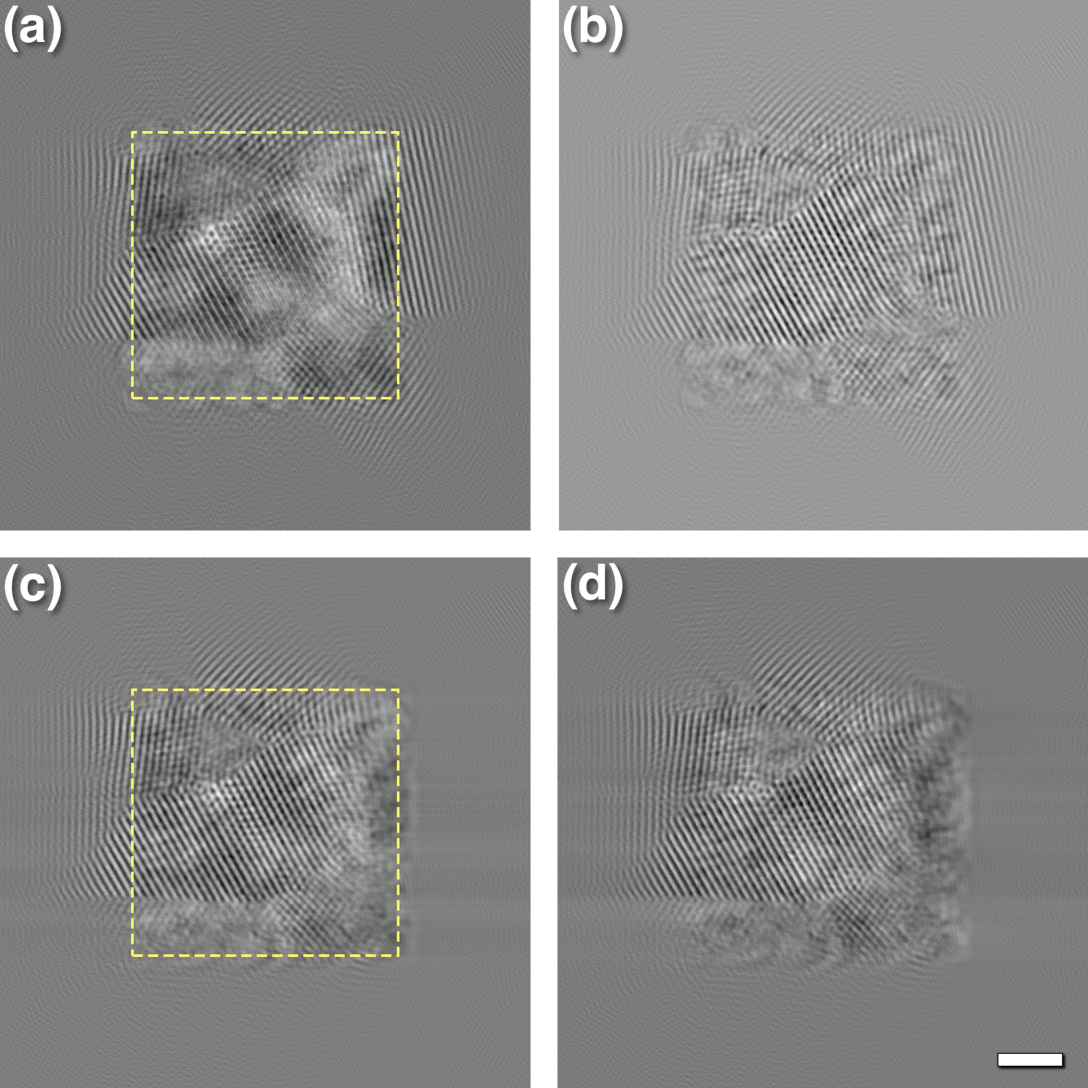
Reconstruction of a tile from a focal series of PtIr with padding. (*a*) Amplitude and (*b*) phase images of a reconstruction of a tile of a focal series padded to twice the size corresponding to the same region as in Fig. 4 ▸(*a*). The yellow outline delimits the part extracted from the original micrograph series before padding. At a defocus of −1660 Å at 200 kV and for the main reflection at 2.25 Å, the average delocalization is ∼18 Å. (*c*) Amplitude and (*d*) phase images of the reconstruction where the right half of the frequency-space representation was eliminated to isolate a side band. The yellow outline denotes the original images before padding, with the delocalized signal recreated to the left of the edge. The reconstruction spills over the edge on the right because of the spread of low-frequency information from the conjugate Ewald sphere, resulting in the blurred appearance. Scale bar: 20 Å.

To accelerate computation and to examine smaller parts of the micrographs, tiles were extracted from the original focal series. However, some of the information at the edges of the reconstruction wrap around because of the delocalization imposed by defocusing, resulting in the ‘twin image’ effect after CTF correction (Downing & Glaeser, 2008 ▸, 2018 ▸). The extracted tile from the focal series was therefore padded to twice its size before parabolic reconstruction. Figs. 6 ▸(*a*) and 6 ▸(*b*) show the amplitude and phase images, respectively, with the extent of the tile indicated by the yellow square in Fig. 6 ▸(*a*). The delocalized signal in the original tile extends far beyond its edge. At a defocus of −1660 Å at 200 kV, the delocalization of the main reflection at 2.25 Å is calculated to be ∼18 Å, comparable to the extent of the signal recovered beyond the edge of the tile. Note that this is only half of the signal, as the delocalization in the other direction was eliminated in extracting the tile. The information inside the tile also lacks information that lies outside the tile boundary.

### 4.5. Single side bands and an alternative view of Ewald sphere correction

Because of an alternative view that the Ewald sphere arises from the combination of electrons scattered in opposite directions (the ‘left–right’ formalism of DeRosier, 2000 ▸), a different handling of the Ewald sphere based on single side bands was proposed and implemented (Russo & Henderson, 2018 ▸). A single side band is obtained by eliminating half of the Fourier transform with a specified angle for the line of elimination. The reconstruction from the padded tile was masked to delete the right side of the Fourier transform (*i.e.* at an angle of 90°) [Figs. 6 ▸(*c*) and 6 ▸(*d*)]. The recovered delocalized signal extends to the left beyond the edge of the original tile, while it is truncated on the right with a blurred part across the edge. The blurred part is likely where the two Ewald spheres overlap, and low-resolution features are retained. Even omitting half of the information, the reconstruction still preserves many features, suggesting that if combined with other images as in cryoEM reconstruction, the coherent part will contribute significantly.

**Figure 7:**
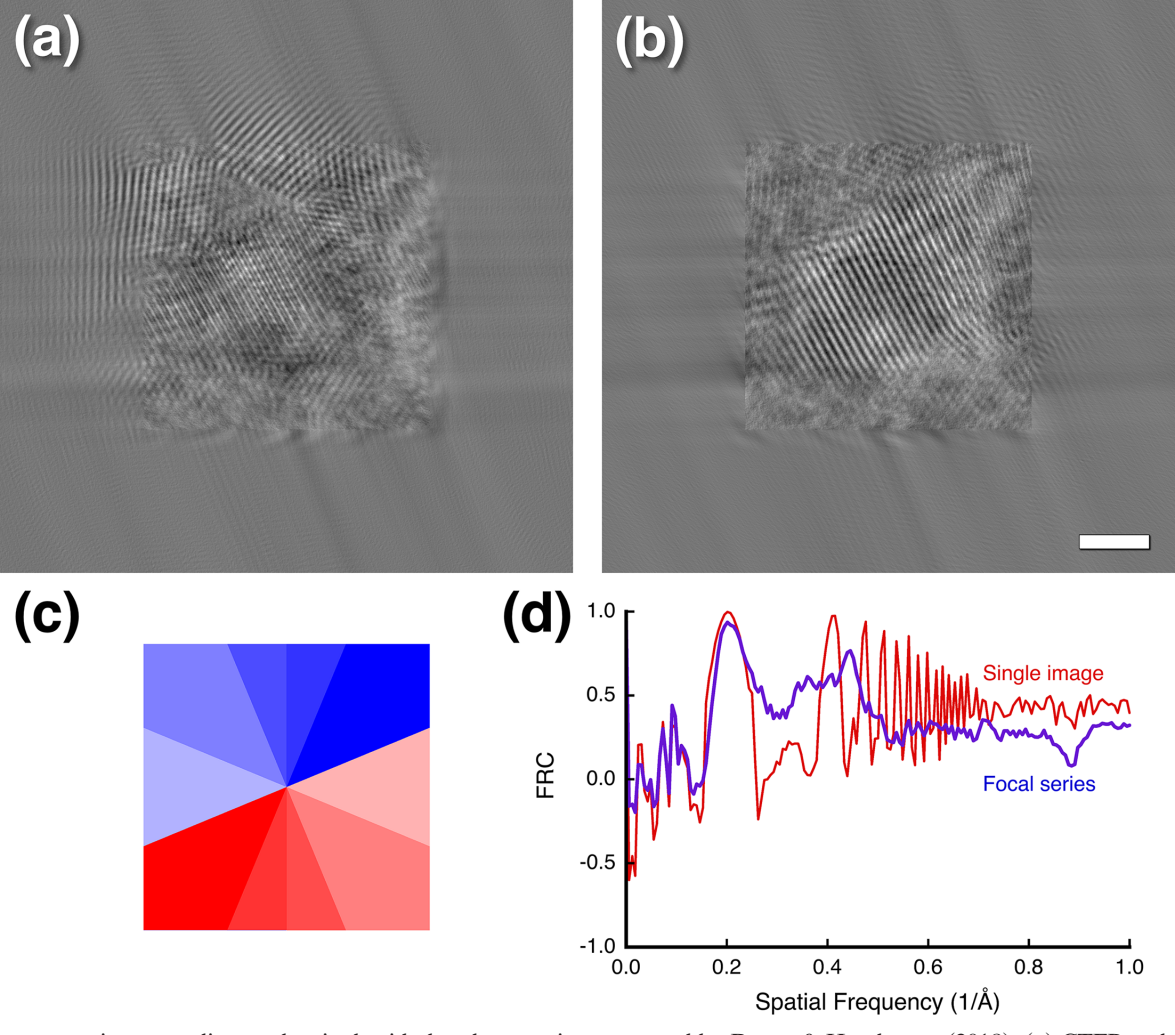
Focal series reconstruction according to the single-side-band correction proposed by Russo & Henderson (2018 ▸). (*a*) CTFP and (*b*) CTFQ focal series reconstruction with padding shows artifacts consistent with the sign of the aberration function shown in (*c*) applied with red positive and blue negative. (*d*) Comparison of the focal series reconstructions of Figs. 6 ▸(*a*) and 6 ▸(*b*) and that in (*a*) here. Scale bar: 20 Å.

While the test in Figs. 6 ▸(*c*) and 6 ▸(*d*) is informative, how would the single-side-band method fare in a focal series reconstruction? The focal reconstruction algorithm was adapted to accurately reflect the originally implemented code with the calculation of the eight segments (Russo & Henderson, 2018 ▸). Figs. 7 ▸(*a*) and 7 ▸(*b*) show the ‘CTFP’ and ‘CTFQ’ reconstructions with obvious artifacts. The oblique streaks are oriented in the direction in which the sign of the aberration function switches [Fig. 7 ▸(*c*)]. The method attempts to alleviate the effect of the abrupt switch in sign by calculating it in the eight sectors. However, Fig. 7 ▸(*c*) shows the sign in the eight sectors, with blue positive and red negative. It therefore does not achieve the desired outcome of avoiding the discontinuity where the sign switches. The consequence is a focal series reconstruction with severe artifacts. The two reconstructed images (CTFP and CTFQ) are not mirror images because the focal series has a direction of focus change.

The main problem with the single-side-band method is the insistence that the CTFP and CTFQ images should have Friedel symmetry (as it is encoded in the MRC image format). Compared with equation (2), it flips the signs of the individual halves of the CTF in half of the image specified by the angle of the line of separation. This then creates a discontinuity that needs to be handled with the sectors approach. Even when calculated in full frequency space with explicit Friedel mates (as in our modified code), the resultant reconstructions are real (no phases). Comparing it with our reconstruction [Figs. 6 ▸(*a*) and 6 ▸(*b*)] we see some correlation [Fig. 7 ▸(*d*)], suggesting that there is partial retrieval of coherent information. Using this method during reconstruction likely enhances only the coherent parts, still resulting in reasonable maps. This means that if a better CTF correction is applied, one should expect to achieve a high resolution from fewer particles than with the single-side-band method.

### 4.6. The phases of the parabolic reconstruction

**Figure 8:**
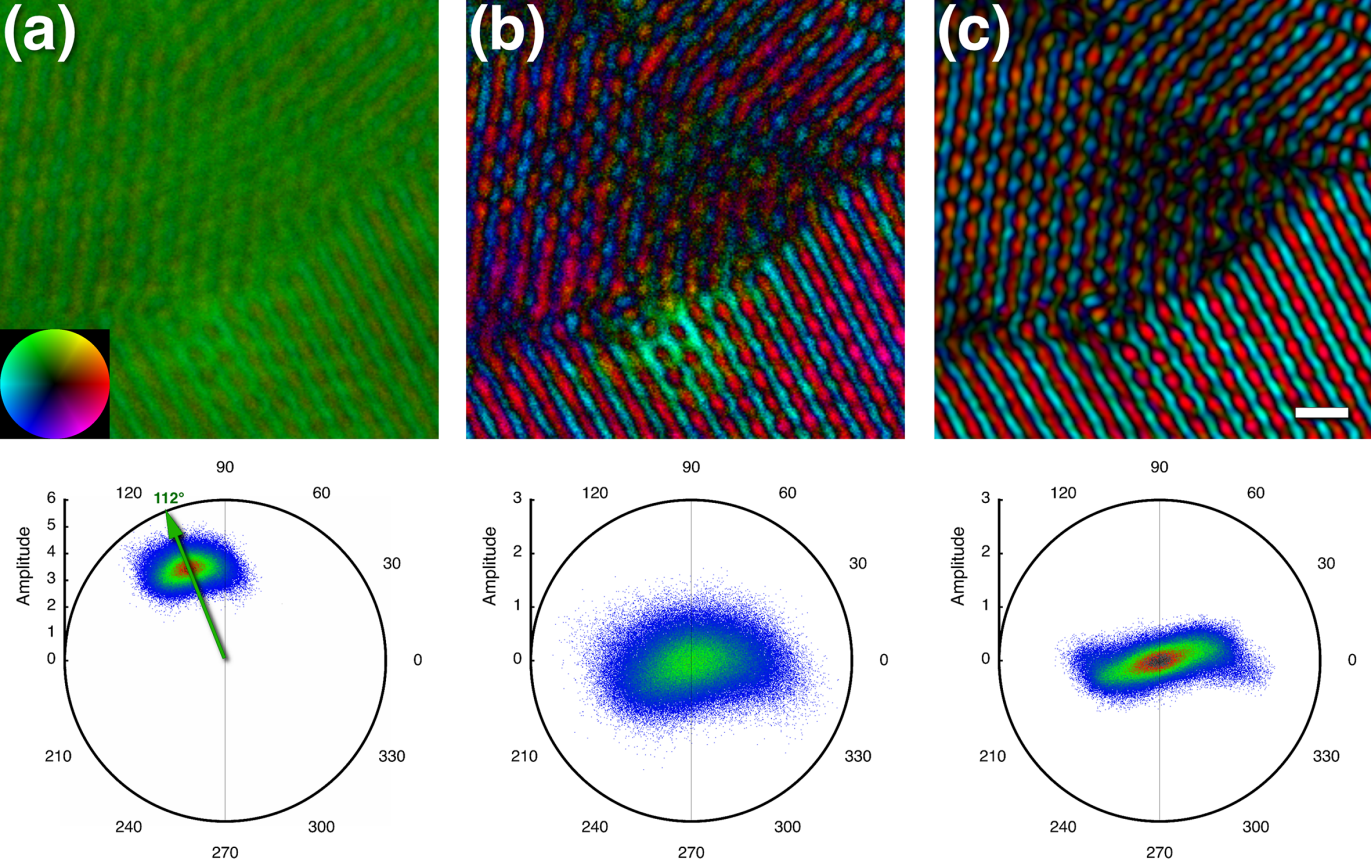
The real-space phases of a parabolic reconstruction of the PtIr specimen. (*a*) The phase-colored amplitude map (top) of the reconstruction shows a clustering of the phases in one quadrant of the polar plot (bottom; also called the complex plane or Argand diagram). The arrow shows that the real-space average phase shift is the sum of π/2 (90°) and the constant phase shift in the CTF of 0.38 (∼22°) used for the Pt amplitude contrast. Inset: phase colors corresponding to the polar plot. (*b*) Filtering out the zero frequency results a better interpretable representation (top) and in a clustering around the origin of the polar plot (bottom). (*c*) Filtering to retain frequencies between 1 and 3 Å gives structural features of the PtIr nanocrystals (top), with the phases peaking close to the real axis in the polar plot (bottom). Scale bar: 5 Å.

While much has been said about the real-space phases in the exit-wave literature, it is not quite clear what they mean. Delocalization of the signal imposed by the CTF and wrapped around the edges of the micrographs may affect the phases. Therefore, the reconstruction of the whole focal series was performed first before extracting a tile away from the edges. The phases in the tile from the reconstruction cover a narrow range peaking at ∼1.95 radians (∼112°) [Fig. 8 ▸(*a*)]. This can be understood as the π/2 shift plus the amplitude contrast that we used in the CTF fitting. Because it mainly affects the zeroth frequency that determines the real-space average, removing it shows the phases distributed around the origin of the polar plot, but with an extension along the real (horizontal) axis [Fig. 8 ▸(*b*)]. Further filtering the frequencies to the 1–3 Å range corresponding to the PtIr nanocrystal reflections narrows the distribution around the real axis [Fig. 8 ▸(*c*)]. The centrosymmetric distribution is probably a reflection of the symmetry in the nanocrystals. The deviation of the phases from 0 and π is likely due to the small offsets of the Ewald spheres from the central plane. The variation in amplitudes in Fig. 8 ▸(*c*) is related to the thickness of the specimen. The crystal in the lower part is much thicker than other two and contributes most to the wings in the polar plot.

The origin of the phases has three components. The first is that the images have largely phase contrast, which means that the images are imaginary, and the phases are shifted by ∼π/2. In addition, the speed of the electrons passing through the specimen slows down because of a significant refractive index, making the amplitude complex. This is typically referred to as ‘amplitude contrast’ and is considered to be mostly constant over the frequency ranges relevant in imaging. Finally, the third component is the phase shift induced by the focal gradient. The first two components are easily removed by omitting the zeroth frequency [Fig. 8 ▸(*b*)]. This is equivalent to subtracting the average from the real-space image, leaving the contrast to oscillate around zero. The remaining phases are then largely determined by the focal change through the specimen [Fig. 8 ▸(*c*)] and deviate little from the real axis. This is particularly evident in Fig. 8 ▸(*c*), where the phase coloring is mostly red (0°) and cyan (180°). This is expected as the Ewald sphere offsets from the central section are small.

### 4.7. Quantitative aspects of a focal series reconstruction

**Figure 9:**
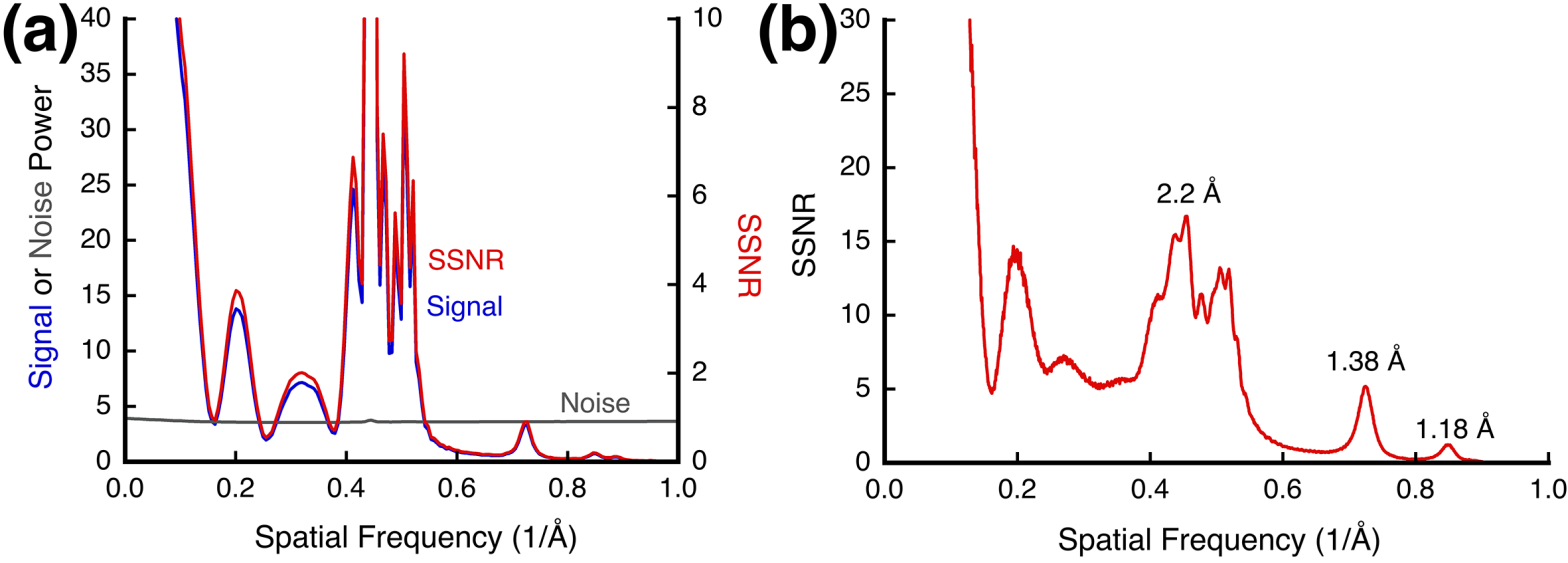
The spectral signal-to-noise ratio (SSNR) for the PtIr specimen. (*a*) The SSNR for one micrograph from the series calculated from a movie taken with a total dose of 3.7 e Å^−2^. The noise spectrum is almost constant because of counting detection, resulting in a close correspondence between the signal and SSNR. The low-frequency spectrum (<0.4 Å^−1^) reflects scattering from the carbon film and the oscillations in the CTF. (*b*) The SSNR from the parabolic reconstruction show the same features but enhanced by *n*^1/2^ = (20)^1/2^ = 4.47. The prominent peaks are associated with the reflections from the PtIr nanocrystals.

To illustrate the averaging process during reconstruction, we can compare the SSNR (spectral signal-to-noise ratio) from the reconstruction with that of a micrograph. Fig. 9 ▸(*a*) shows the SSNR calculated for the central micrograph movie in the series according to the method of Heymann (2022 ▸). We also estimated the radiation tolerance of the specimen (Supplementary Section S6), concluding that at the low dosages we use the PtIr nanocrystals are effectively undamaged. The low-frequency part of the spectrum up to ∼2.5 Å displays the oscillations from the carbon film that was used in CTF fitting [compare with Fig. 2 ▸(*a*)]. The higher frequencies exhibit the reflections from the PtIr nanocrystals. Fig. 9 ▸(*b*) shows the SSNR from the parabolic reconstruction with the corresponding features. The minima for the CTF oscillations are now less evident because of the averaging over a focal range. The integration over a set of images increases the SNR by a factor of *n*^1/2^ (Kirkland *et al.*, 1980 ▸; Saxton, 1994 ▸). The SSNR of the reconstruction in Fig. 9 ▸(*b*) is therefore enhanced by an approximate factor of (20)^1/2^ = 4.47 over that of a single micrograph [Fig. 9 ▸(*a*)]. The reconstruction could potentially be improved beyond the highest reflections at 1.18 Å by better micrograph alignment and aberration function refinement. Nevertheless, these experiments demonstrate that the parabolic reconstruction preserves the same quantitative properties as for micrograph movies (Heymann, 2022 ▸) and cryoEM reconstructions (Heymann, 2019 ▸).

Reconstructions from focal series typically involve compensation for the suppression of information around the minima in the CTF (Schiske, 1968 ▸; Frank, 1972 ▸; Saxton, 1994 ▸; Duden *et al.*, 2014 ▸). In addition, it also attempts to adjust for the envelope functions attenuating high-resolution information (Coene *et al.*, 1996 ▸; Op de Beeck *et al.*, 1996 ▸). The unfortunate consequence is that any noise is also amplified, rendering the adjustment suspect and potentially introducing artifacts. It also affects the quantitative information in the micrographs, changing the relative contributions of signal and noise. I therefore avoid such adjustments and retain the quantitative information inherent in the images without rescaling (Fig. 9 ▸).

### 4.8. Iterative refinement of the reconstruction against the original images

**Figure 10:**
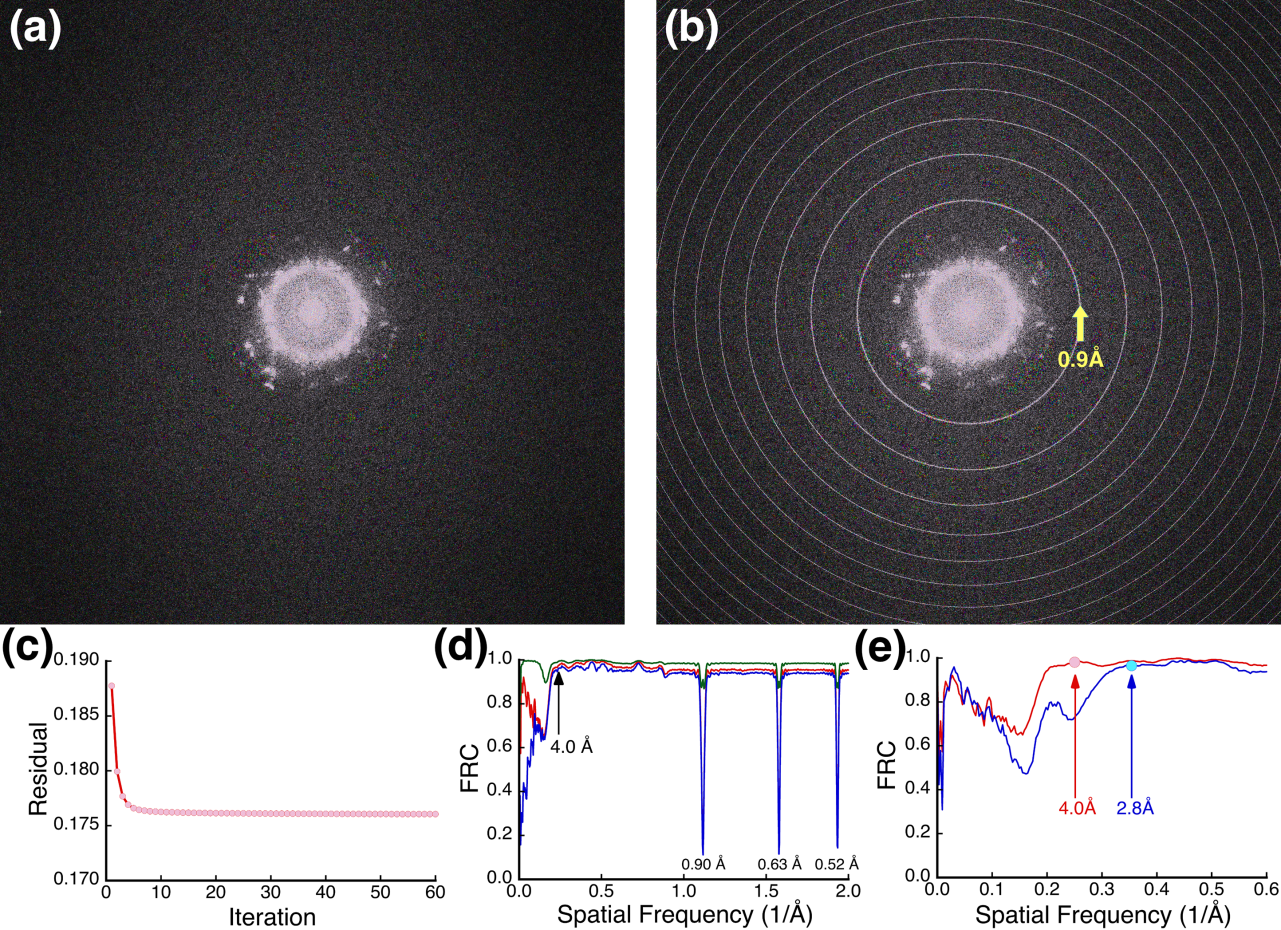
Reconstruction of a PtIr focal series. (*a*) Power spectrum of a parabolic reconstruction. (*b*) Power spectrum of an iteratively refined parabolic reconstruction (1000 iterations). (*c*) Root-mean-square deviation of amplitudes of the iterative reconstruction refinement compared with the original intensities. (*d*) Fourier ring correlation (FRC) between the parabolic reconstruction and the refined reconstruction after 4 (green), 60 (red) and 1000 (blue) iterations. The iterative refinement recovers amplitudes at low frequencies (<1/4 Å), but introduces artifacts where the spheres intersect the Nyquist frequency in the 3D context indicated by the rings in (*b*) and the minima in (*d*). (*e*) FRC as in (*d*) for reconstructions (60 iterations) from a focal range of 640 Å (red) or 320 Å (blue), indicating the difference at frequencies below the points where the Ewald spheres separate at (640 Å × 0.025 Å)^1/2^ = 4 Å (red dot) and (320 Å × 0.025 Å)^1/2^ = 2.8 Å (blue dot), respectively.

The parabolic reconstruction recreates only part of the original images. Fig. 10 ▸(*a*) shows a power spectrum of the reconstruction, with reflections beyond 2 Å with no conjugate counterpart. This deviation from Friedel symmetry is expected from the information on the Ewald sphere. I adopted the conceptual method of Allen *et al.* (2004 ▸) to refine the reconstruction against the intensities of the original images.^1^ Fig. 10 ▸(*b*) shows the power spectrum from the refined reconstruction, and Fig. 10 ▸(*c*) follows the correspondence with the intensities in the original images. The refinement changes most within the first four iterations, as reported previously (Allen *et al.*, 2004 ▸), followed by slow convergence. The power spectrum [Fig. 10 ▸(*b*)] and the comparison with the original parabolic reconstruction [Fig. 10 ▸(*d*)] indicate artifacts as rings in the power spectrum. These correspond to where the spheres wrap around in the *z* direction in the 3D transform and reflect a discontinuity in the phases [the first is at (0.025 Å × 32 Å)^1/2^ = 0.93 Å]. The other feature in the FRC comparison [Fig. 10 ▸(*d*)] is an increasing disagreement at low frequency with iterations. The inflection for this is a function of the focal range [*s* = (1/λΔ*f*_r_)^1/2^], as illustrated in Fig. 10 ▸(*e*), where the blue curve is for a reconstruction performed with ten middle images rather than the full 20 images in the focal series. Visual comparison of the parabolic reconstruction and its iterative refinement shows little difference, likely because the low frequencies do not significantly contribute to the PtIr nanocrystal structure and very high frequencies are dominated by noise. I conclude from these experiments that refinement against the original images recovers some of the information from the second Ewald sphere, while it introduces artifacts in the reconstruction at the same time. The straightforward parabolic reconstruction [equation (15)] is therefore a better representation of one Ewald sphere at higher frequencies than the iterative reconstruction.

## 5. Discussion

Our understanding of image formation in the electron microscope is essential to developing the appropriate algorithms for cryoEM reconstruction. Therefore, focal series of electron micrographs were analyzed to better understand image formation and how it relates to theory. The pertinent outcomes inform on the validity of the theory and how that can lead to a better reconstruction approach.

### 5.1. The assignment of the two parts of the CTF to the two Ewald spheres is justified

In the linear image-formation model, the spherical scattering by the specimen and the subsequent focusing correspond to the conjugate Ewald spheres [Fig. 1 ▸(*a*)] (Heymann, 2023 ▸). These spheres coincide with those visualized by the 3D Fourier transform of a focal series that mimics the focal gradient (Fig. 2 ▸; Taniguchi *et al.*, 1991 ▸; Kimoto *et al.*, 2012 ▸, 2013 ▸; Ishizuka & Kimoto, 2016 ▸). In the case of GO, simply shifting the six reflections on the Ewald sphere to the central section corrected for the focal gradient (Fig. 3 ▸). Note that the narrowness of the spheres in the 3D transform is a reflection of the focal range, while the Ewald spheres arising from GO itself extend much futher.

With the strongly scattering PtIr specimen, we have much more information on the spheres in the 3D transform [Fig. 5 ▸(*b*)], and its analysis illustrates the correct interpretation of the CTF. Correcting the CTF with the form for the projection approximation [equation (1)] produces a 3D power spectrum with a central plane and two spheres with curvature twice that of the original spheres [Fig. 5 ▸(*d*)]. In contrast, the application of only one half of the CTF to the focal series flattens one sphere while doubling the curvature of the other [Fig. 5 ▸(*e*)]. Applying the conjugate function flattens the other sphere [Fig. 5 ▸(*f*)]. Therefore, the application of equation (1) leads to the superposition of the two halves of the CTF and the limitation ascribed to the Ewald sphere in the projection approximation.

The conclusion is thus that the proper way to correct the micrographs is to apply the halves of the CTF separately. Correcting the micrographs with one half of the CTF and calculating the 3D transform of the corrected stack yields the equivalent of the parabolic reconstruction in the central section [Fig. 1 ▸(*b*)]. Iterative refinement of the parabolic reconstruction recovers information from the second sphere at low frequencies, while retaining the information beyond the separation of the spheres [Figs. 10 ▸(*d*) and 10 ▸(*e*)]. This is fully consistent with the assumptions made for equation (2) and the model of the two Ewald spheres relating to the two halves of the CTF.

### 5.2. The upper Ewald sphere is the frequency-space representation of the exit wave in real space

The exit wave is commonly interpreted as the pattern of scattered electrons emerging from the bottom of the specimen. It should have real-space phases that reflect the shifts introduced by scattering. In the literature, any method that produces phases has been claimed to reconstruct the ‘exit wave’. Focal series reconstructions have commonly been used to calculate such phases, either directly (as was performed here; Duden *et al.*, 2014 ▸; Biran *et al.*, 2022 ▸) or iteratively (Schiske, 1968 ▸, 2002 ▸; Frank, 1972 ▸; Kirkland *et al.*, 1980 ▸; van Dyck *et al.*, 1996 ▸; Op de Beeck *et al.*, 1996 ▸; Ishizuka, 2013 ▸; Ming *et al.*, 2018 ▸; Huang *et al.*, 2021 ▸; Zhang *et al.*, 2024 ▸). Questions about whether the iterative methods yield unique answers have been raised (Ferwerda, 1988 ▸). From Fig. 10 ▸ it is evident that iterative refinement reintroduces information from the second Ewald sphere. Of note is that most of these studies focus on crystals with small unit cells, where the reflections are beyond the overlap of the Ewald spheres. Therefore, the desired results are not really influenced by the iterative process, and the information of interest lies on one Ewald sphere. In cryoEM reconstructions at lower frequencies where the Ewald spheres overlap the correction is simply according to equation (1).

What is evident from Fig. 8 ▸ is that the phases depend on the CTF parameters. The π/2 and amplitude contrast contributions affect only the zeroth frequency and thus the shift in contrast in real space. The structural information is contained in the rest of the phases. The Ewald spheres represent a small deviation from the central section in the 3D transform, which means that the associated changes in phases should be small. Fig. 8 ▸(*c*) indeed shows that this is the case, with most of the phases close to 0° and 180°. The conclusion is that the correction of an image by one half of the CTF that corresponds to the upper Ewald sphere yields the exit wave. This is also true for each image in the focal series, with the summation serving to increase the SNR (Fig. 9 ▸). In cryoEM reconstruction, correction of a particle image with one half of the CTF produces the information on the corresponding Ewald sphere, and the conjugate half on the conjugate Ewald sphere.

### 5.3. Only single scattering contributes to the Ewald spheres

A common interpretation is that the sphere ridges of the 3D transform of a focal series represent the linear parts, with additional nonlinear parts distributed over the whole 3D Fourier transform (Op de Beeck *et al.*, 1996 ▸; Han *et al.*, 1995 ▸; Kimoto *et al.*, 2013 ▸). The nonlinear parts have been shown to be a function of the energy variation in the source (Coene *et al.*, 1996 ▸). Thus, with a highly coherent beam, these nonlinear contributions are negligible and only the linear parts contribute to the coherent signal, while the rest is averaged out as noise (*i.e.* reduced by *n*^1/2^). The focal series imposes a hat function on the beam direction so that the coherent signal is confined to a sinc function in frequency space with a width of 2/focal range (∼2 pixels in the 3D transform; Fig. 2 ▸). Similarly, the integration over the *z* coordinates of the specimen confines the coherent signal to a sinc function (with a different width). Therefore, only in the case for graphene is there an extension of the signal in the beam direction, whereas for any thicker specimen the width of the Ewald sphere is narrower. Because this can all be demonstrated with a single elastic scattering model [equation (2)], there is little reason to believe that any other contributions are of significance in reconstruction.

### 5.4. Correcting for the CTF in cryoEM reconstructions

The most common correction for the CTF is based on equation (1). The achievement of very high resolution reconstructions (wwPDB Consortium, 2024 ▸) indicates that the signal coherence over the thousands of particle images used is adequate, as long as reconstruction is consistent with the Ewald sphere (Heymann, 2023 ▸). However, equation (9) suggests an improvement in correcting for the CTF. Integrating each Ewald sphere into the reconstruction volume separately reflects equation (1) where the two spheres overlap and equation (2) where they separate. The only remaining question is the ambiguity of which Ewald sphere is which. In principle, this can be resolved by performing reconstructions in both ways and assessing the final resolution. It is likely that the correct hand can be determined from the proper CTF correction and considering any image mirroring in processing. In terms of reaching better reconstruction resolutions, the expectation is that the typical overdetermination of the problem by picking a very large number of particle images will impose sufficient coherence so that correct handling of the Ewald sphere will not necessarily lead to improvements. The distinction would rather be more evident at smaller numbers of particles, as is the case for other parameters (Heymann, 2015 ▸, 2019 ▸).

Finally, caution is recommended in thinking that the Ewald sphere phases used for reconstruction can be improved by some reference or iterative method adjusting phases either in real or frequency space. In the noise-dominated micrographs this is more likely to corrupt the phases than improve the reconstruction (Wolf *et al.*, 2006 ▸).

## 6. Conclusion

I have presented the theory and some experiments to show that the parabolic reconstruction from a focal series of micrographs is the closest representation of one Ewald sphere and is consistent with the concept of an exit wave. Everything derives from the linear image-formation model as expressed in equation (2). With it we can simulate the spherical curves observed in the 3D Fourier transform of a focal series. It indicates that we can calculate the information of one Ewald sphere by imposing one part of the CTF and amplifying the SNR by parabolic reconstruction. It also maintains that the recorded micrographs are phase-free because of the superposition of the two conjugate Ewald spheres. Interpreting the complex real-space images resulting from the parabolic reconstruction is not trivial and would require further investigation. The theory presented here clarifies the reason why we obtain high-resolution reconstructions in cryoEM when we integrate particle data along the Ewald spheres. Even if this linear model of image formation is not exact, it offers a very workable model that is highly applicable in cryoEM.

## Supplementary Material

Additional information regarding CTF fitting of a focal series, more examples of focal series 2D and 3D power spectra, more reconstruction details and radiation sensisitivity of the PtIr specimen. DOI: 10.1107/S2052252525010796/eh5024sup1.pdf

## Data Availability

The original data are available on request. *Bsoft* is available at https://bsoft.ws.

## Footnotes

1 Whether we use amplitudes or intensities in the iterative refinement does not affect the qualitative results, just a moderate quantitative adjustment of the final intensities.
