# Supplementary material for: The relationship between the Ewald sphere and exit wave explored using focal series electron micrographs
Source: IUCrJ. 2026 Jan 1;13(Pt 1):77–93. doi: 10.1107/S2052252525010796 (PMC12809427; doi:10.1107/S2052252525010796)
Supplement: Supplementary file 1 [file m-13-00077-sup1.pdf]

IUCrJ

Volume 13 (2026)

Supporting information for article:

The relationship between the Ewald sphere and exit wave explored using focal series electron micrographs

J. Bernard Heymann

S1. Determining the CTF parameters from a focal series

Fitting the CTF of a focal series benefits from more information than a single image. I therefore wanted to examine the possibility of extending the CTF to more than the major parameters presented in the main text. I therefore extended the fit to other symmetric terms (Table S1). For this I write the CTF phase shift as the polynomial series:

$$\gamma(s,\phi) = \frac{2\pi}{\lambda}\left[c_0 + \sum_{n=1}^{N} \frac{(\lambda s)^n}{n} \sum_{m=-n}^{n} c_{n,m} b^m(\phi)\right] \quad \text{(S1)}$$

Where λ is the electron wavelength, s is the spatial frequency ($s = \sqrt{u^2 + v^2}$), ϕ is the radial direction ($\phi = \tan^{-1}\frac{v}{u}$), the iteration step for subscript m is 2 (i.e., m+n is even), and the basis functions are:

$$b^m(\phi) = \begin{cases} \sin -m\phi, & m < 0 \\ 1, & m = 0 \\ \cos m\phi, & m > 0 \end{cases} \quad \text{(S2)}$$

The scattering angle is given by $\theta = \lambda s$ in the small angle approximation. Note that the coefficients $c_{n,m}$ are independent of the wavelength and has a unit of length. The label n is also referred to as the radial degree and the label m as the azimuthal degree. For fitting it is convenient to convert the coefficients to wavelength dependent weights:

$$w_0 = \frac{2\pi}{\lambda} c_0$$

$$w_{n,m} = 2\pi \frac{\lambda^{n-1}}{n} c_{n,m} \quad \text{for n>0} \quad \text{(S3)}$$

The major terms of the CTF are now: w_0 is the amplitude contrast, $\Delta f = c_{2,0}$is the average defocus, the deviation in defocus (astigmatism) is given by the two terms $c_{2,-2}$ and $c_{2,2}$, and the spherical aberration is $C_s = c_{4,0}$. From Table S1 I conclude that the fourth order terms other than the spherical aberration are negligible.

Table S1 Objective lens aberration parameters determined from focal series of thick carbon (3 series), graphene oxide (two sets of 4 series each taken on different days and one tilted series) and platinum-iridium on carbon film (two sets of 3 series, one with a 10 eV slit and one without energy filter).

Parameter	Unit	Carbon	Graphene oxide			Platinum iridium	
			Set 1	Set2	Tilted	10 eV slit	No slit
$w_{0,0}$	radians	0.15	0.10	0.11	0.13	0.38	0.38
		(0.05)	(0.008)	(0.012)		(0.003)	(0.006)
$c_{0,0}$	pm	-0.06	-0.04	-0.04	-0.05	-0.15	-0.15
		(0.02)	(0.003)	(0.005)		(0.001)	(0.003)
$c_{2,-2}$	nm	-8	-21	10	-1204	-1	-0.3
		(15)	(48)	(9)		(2)	(0.3)
$c_{2,0}$	nm	-709	-2323	-1278	-2160	-1496	-1542
		(20)	(1954)	(847)		(117)	(65)
$c_{2,2}$	nm	-46	12	47	342	-11	-17
		(27)	(72)	(3)		(4)	(3)
$c_{4,-4}$	mm	-0.007	-0.0001	-0.001	0.001	0.001	0.001
		(0.009)	(0.0002)	(0.001)		(0.001)	(0.001)
$c_{4,-2}$	mm	-0.001	-0.0001	-0.004	0.001	-0.001	0.001
		(0.002)	(0.0005)	(0.006)		(0.001)	(0.001)
$c_{4,0}$	mm	2.50	2.68	2.54	2.65	2.63	2.56
		(0.07)	(0.10)	(0.01)		(0.10)	(0.12)
$c_{4,2}$	mm	0.002	0.003	0.001	0.003	0.001	0.001
		(0.005)	(0.005)	(0.002)		(0.001)	(0.001)
$c_{4,4}$	mm	0.007	0.005	0.001	0.001	-0.001	-0.001
		(0.005)	(0.006)	(0.003)		(0.001)	(0.001)

Standard deviation in brackets

S2. Testing the interdependence of CTF parameters

The power spectra of focal series of graphene oxide (GO) and platinum-iridium (PtIr) on carbon film were calculated and the CTF fitted by hand in the Bsoft program **bshow**. One set of fits was done with the nominal parameters assumed for the microscope, namely amp=0.1 (amplitude contrast) and Cs=2.7 mm (spherical aberration) (Figure S1a). A second set was done with different values: amp=0.5, Cs=2.2 mm (Figure S1b). Figure S1 shows that these two sets of fits consistently differ in defocus ~230 Å for GO and ~220 Å for PtIr. The conclusion is that the absolute value of defocus is unreliable because we do not have accurate measures of the amplitude contrast and spherical aberration.

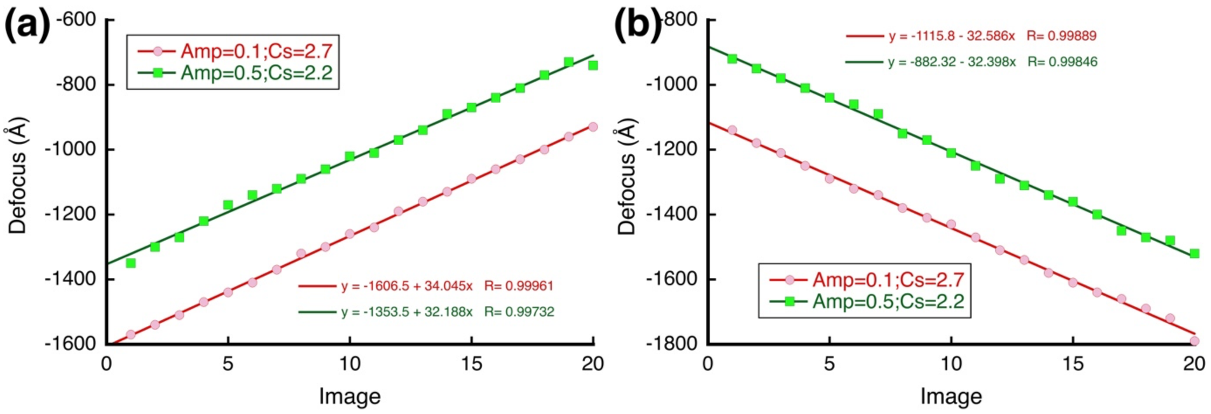

Figure S1: CTF manually fit to power spectra from a focal series of (a) graphene oxide and (b) platinum-iridium on carbon support. In each graph the two curves correspond to fits with different amplitude contrast and spherical aberration coefficients. It shows that the focus values obtained are sensitive to these parameters. While the focal series was taken at a nominal 37 Å focus step size, the actual step size is closer to 32-34 Å.

To further illustrate the interdependence of the CTF parameters, I calculated CTF curves with different parameters but fitted to show the same oscillations over part of the spectrum (Figure S2). I can readily produce sets of parameters that show good correlation at high frequencies. The distinction is at low frequencies that are often obscured in large features in electron micrographs, involving very small angle scattering such as inelastic scattering. Because most of the emphasis is on fitting the high resolution parts of the spectrum, the fitted parameters are adequate to model the CTF oscillations for a reasonable level of correction. Therefore, even if we cannot determine defocus accurately, the relative defocus with respect to other parameters are sufficient to support reconstruction of high resolution details.

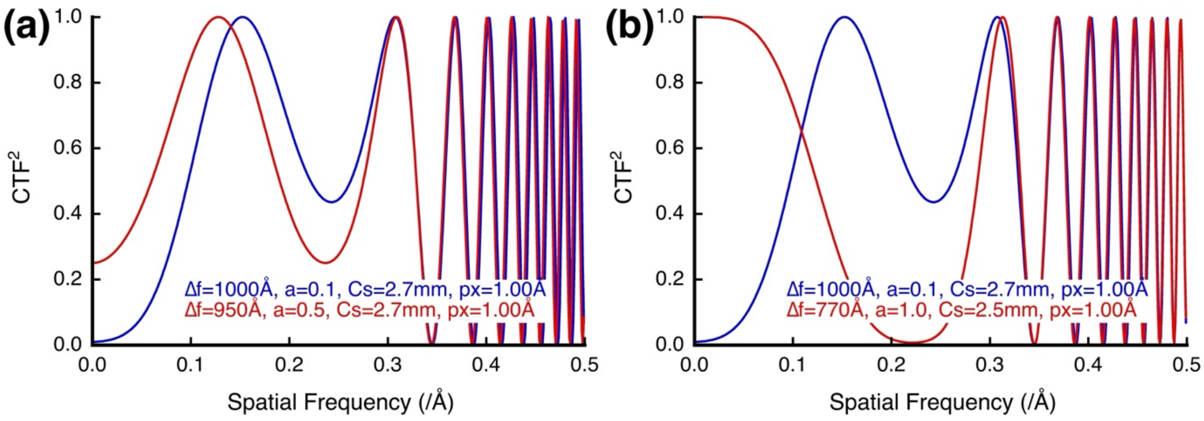

Figure S2: Choices for amplitude contrast ($a = w_{0,0}$) and the spherical aberration coefficient ($C_s = c_{4,0}$) can be offset by defocus ($\Delta f = c_{2.0}$), giving a good correspondence at high frequencies. It is only at low frequencies where the effect of amplitude contrast is clear, typically obscured by inelastic scattering in real micrographs.

S3. Focal series from carbon film and single layer graphene oxide

Focal series of carbon film and graphene oxide were collected as described in the main text. Orthogonal views through the stack of 2D power spectra from the carbon film show the distinct oscillations of the Thon rings, with the progression through the focal changes in the transverse orthogonal views (Figure S3a). The curved shapes are characteristic of the spherical aberration included in the CTF. In the 3D power spectrum of the stack of micrographs, the spherical curves in the transverse views are only one pixel thick, as is expected of the width of the sinc function related to the focal range (Figure S3b).

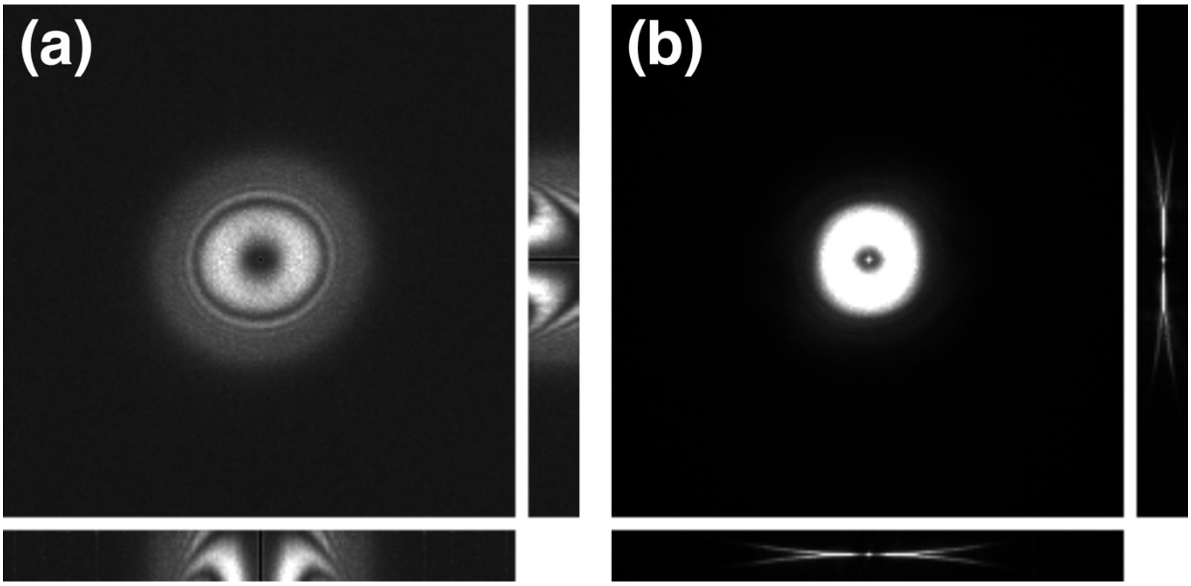

Figure S3: Thick carbon (~400 Å). (a) Orthogonal views of a stack of 2D power spectra of micrographs near true focus, showing the change in the contrast transfer function in the lateral views. (b) Orthogonal views of the 3D power spectrum of the same stack of micrographs, with the coherent spheres resulting from the focus gradient evident in the lateral views.

The 2D power spectra of a stack of images from a focal series of graphene oxide exhibit the six primary reflections at 2.13 Å, as well as a faint background with the oscillations of the CTF (Figure S4a,c). The insets show the oscillations in the z direction where the period relates to the offset of the Ewald sphere from the central section. In the 3D transforms of the focal series (Figure S4b,d) the splitting of the reflections onto the two spheres is shown in the insets.

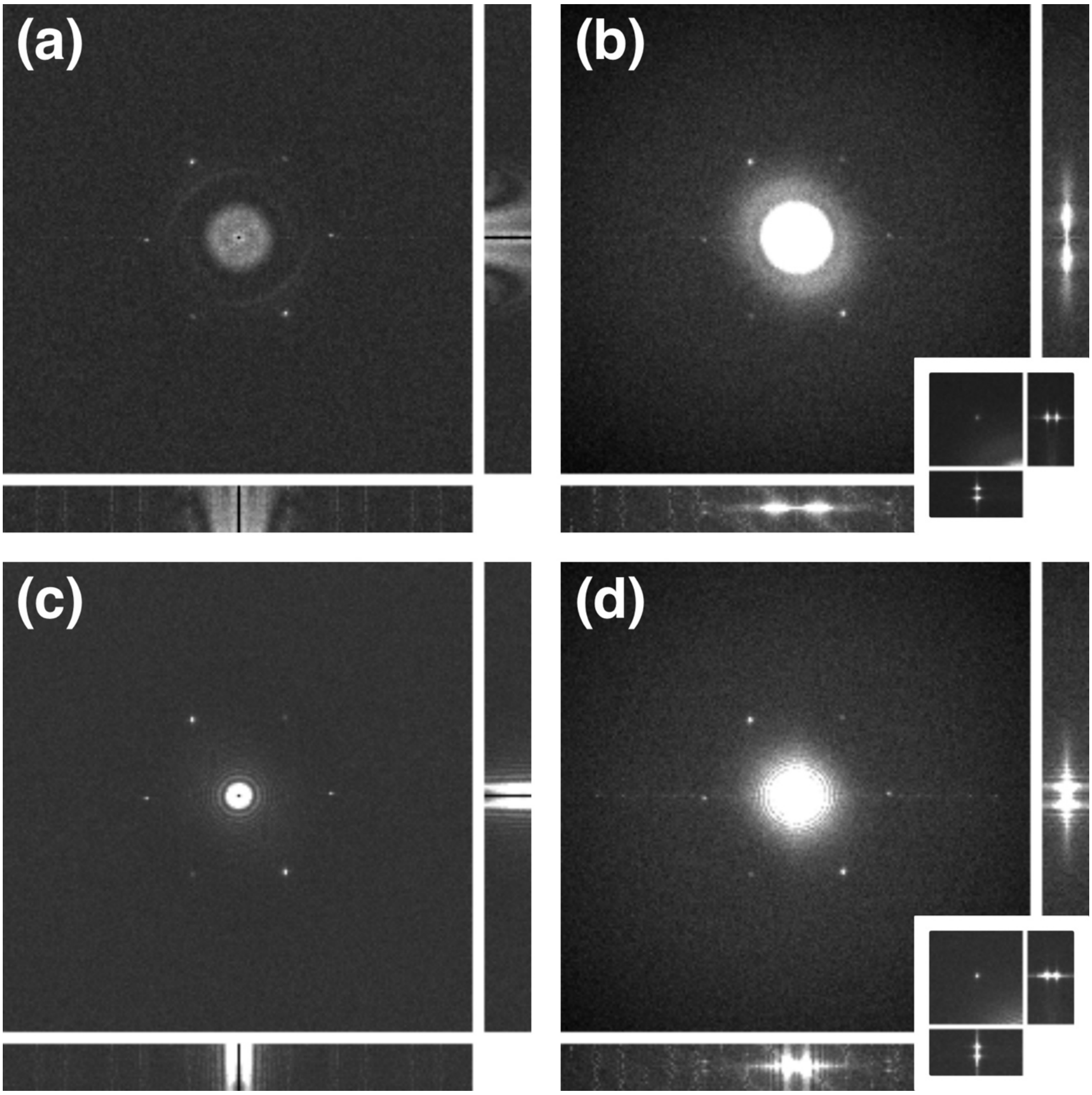

Figure S4: Orthogonal views of a thin layer of graphene oxide near true focus (a,b) and defocused ~0.6 µm (c,d), showing the 2D power spectra of the focal series (a,c) and the 3D power spectra (b,d). The insets show the top, left diffraction spot at 2.13 Å. In the 2D series, the spot intensity shows a periodic variation that reflects the offset of the sphere. In the 3D power spectrum, the two spots fall on the two spheres, offset to a frequency of 0.0027/Å. The vertical artifacts likely result from inadequate gain correction.

S4. Energy filtering of the PtIr specimen

We collected focal series of the PtIr specimen with and without the energy filter. The power spectra of two focal series of the same area, one with a 10 eV slit and the other with the slit wide open, are almost identical at lower frequencies, with a small divergence at higher frequencies (Figure S5). This is likely the result of the elimination of inelastically scattered electrons that is present in the unfiltered images as high frequency noise.

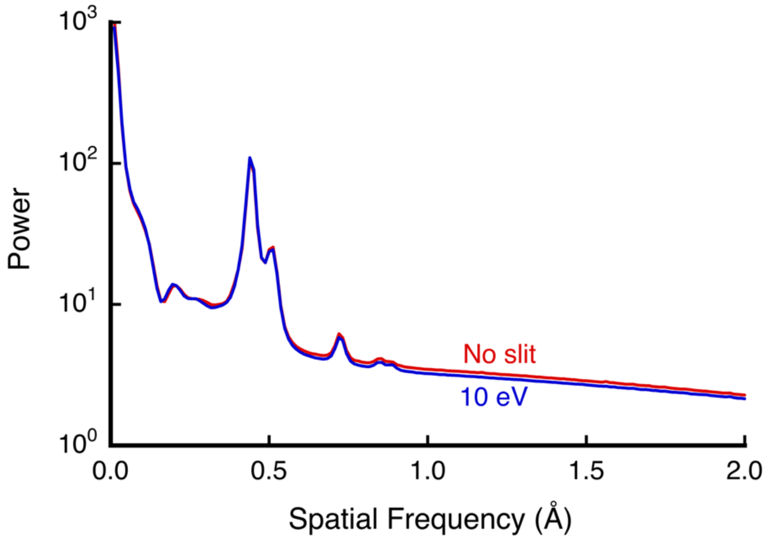

Figure S5: Average power spectra of focal series of the PtIr specimen with a 10 eV energy filter slit (blue) compared with the slit open (red) differs slightly in the high frequencies where noise dominates.

S5. Parabolic reconstruction of the PtIr specimen

The parabolic reconstruction was performed using the fitted CTF parameters, yielding a complex image. Figure S6 shows the different representations extracted from a bigger reconstruction. Because the phases associated with the PtIr nanocrystals are mostly distributed along the real axis (see Figure 7c in the main text), the detail in the phase image (panel d) corresponds best to the real image (panel a), while the imaginary and amplitude images reflect intensity variations (panels b and c) likely corresponding to varying thickness.

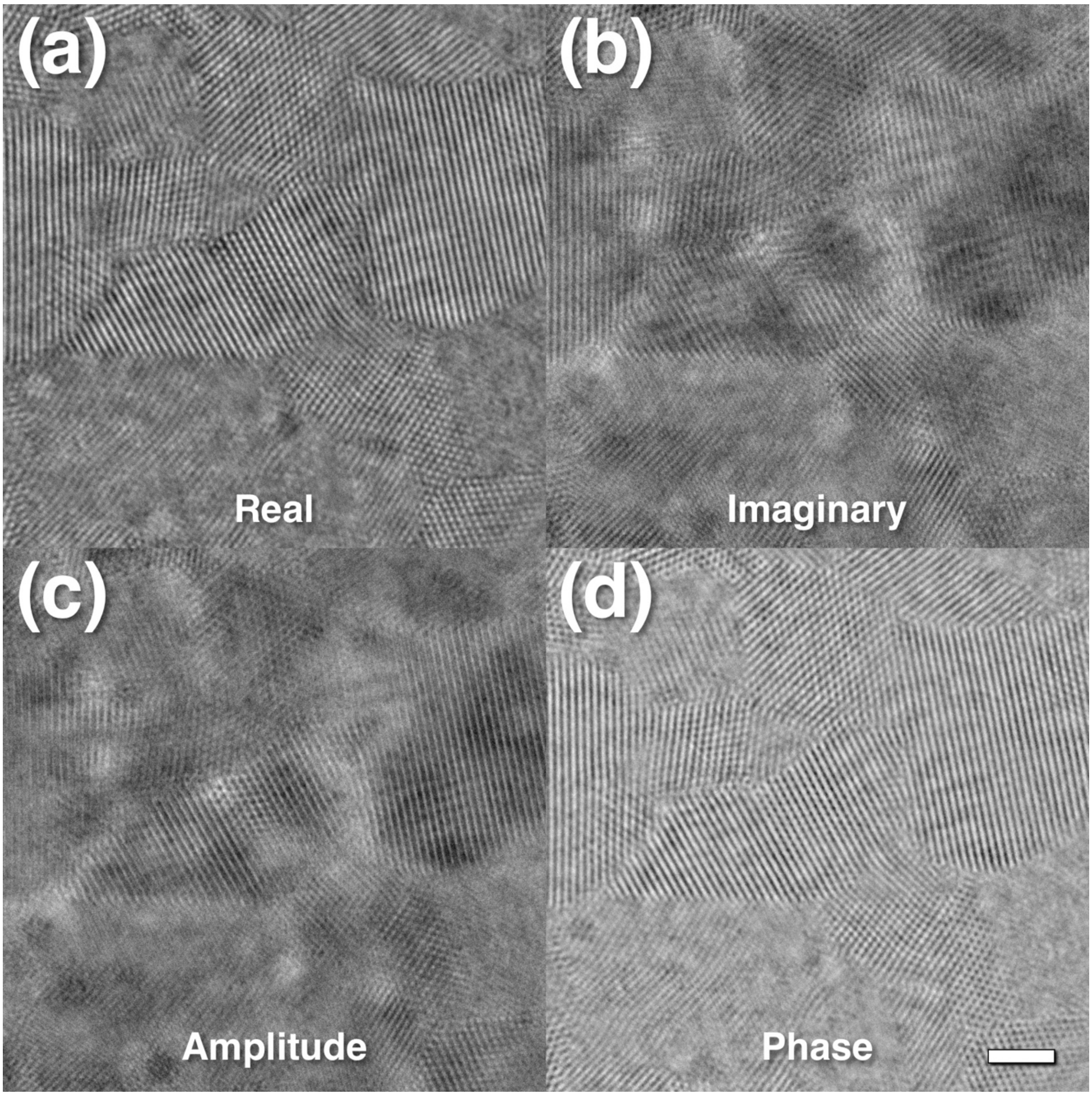

Figure S6: Part of a parabolic reconstruction from a focal series of micrographs of PtIr. The panels show the (a) real, (b) imaginary, (c) amplitude and (d) phase representations of the reconstruction. Scale bar: 20 Å.

S6. Radiation sensitivity of the PtIr specimen

I calculated the power accumulated after intervals of 8 frames within a movie series using the program **bseries**. The SSNR accumulates linearly with dose (Figure S7), indicating high dose tolerance. I also assessed the change in the crystalline reflection intensities of peaks in the CTF at difference spatial frequencies. These intensities vary little and at low frequencies even increase slightly. It therefore appears that there may be a very small beam-induced ordering. Only at high frequencies do the intensities decrease slightly indicating possible damage. I conclude that at such a low accumulated dose of ~80 e/Å^2 for a focal series the specimen is effectively undamaged.

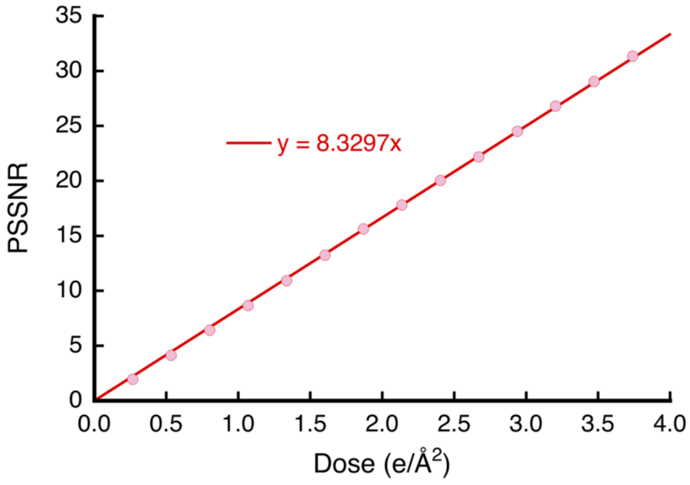

Figure S7: Progressive signal-to-noise ratio for the PtIr specimen with a 10 eV energy filter slit at a spatial frequency of 0.45/Å (2.2Å), the major peak in the diffraction pattern.
